# *BTN2A1* and *BTN3A1* as Novel Coeliac Disease Risk Loci: An In Silico Analysis

**DOI:** 10.3390/ijms262110697

**Published:** 2025-11-03

**Authors:** Kim Ngan Luu Hoang, Shelley Evans, Thomas W. Willis, Kate Davies, Hannah Kockelbergh, Lee Silcock, Kim Piechocki, Anna Fowler, Elizabeth J. Soilleux

**Affiliations:** 1 Department of Pathology, University of Cambridge, Cambridge CB2 1TN, UK; 2 Medical Research Council Biostatistics Unit, University of Cambridge, Cambridge CB2 1TN, UK; tw395@cam.ac.uk; 3 Center for Immunity and Immunotherapies, Seattle Children’s Research Institute, Seattle, WA 98105, USA; 4 Division of Infection and Immunity, School of Medicine, Cardiff University, Cardiff CF14 4YS, UK; 5 Department of Health Data Science, University of Liverpool, Liverpool L69 7ZX, UK; 6 The Kennedy Institute of Rheumatology, University of Oxford, Oxford OX1 2JD, UK; 7 Nonacus Ltd., Quinton Business Park, Birmingham B32 1AF, UK

**Keywords:** coeliac disease, butyrophilin family, hypervariable region 4 (HV4), UK Biobank

## Abstract

Coeliac disease (CeD) is a gastrointestinal enteropathy triggered by the consumption of gluten in predisposed individuals. A recent study showed that individuals were at more than 10% risk of having CeD if a first-degree relative also had the disease. However, only around 50% of CeD genetic heritability is attributable to specific loci, with the majority of this heritable risk attributed to the *HLA* loci, while the remaining 50% of disease risk is currently unidentified. We investigated the butyrophilin family of immunomodulators as novel CeD risk loci. We sequenced the butyrophilin loci of 48 CeD and 46 control patients and carried out gene-based burden testing on the captured single-nucleotide polymorphisms (SNPs). We found a significantly increased *BTN2A1* gene burden in CeD patients. To validate these results, the SNP data of 3094 CeD patients and 29,762 control participants from the UK Biobank database were subjected to single-variant analyses. Fourteen *BTN2A1*, ten *BTN3A1,* and thirteen *BTN3A2* SNPs were significantly associated with CeD status. These results are interesting, as *BTN2A1* and *BTN3A2* have not been associated with CeD risk previously but are known to modulate the activation of Vγ9+ γδ T cells and NK cells. Twenty of the 37 SNPs above were associated with CeD status independent of the risk-associated *HLA* genotypes. All twenty of these SNPs, alongside a novel SNP not included in the above SNPs, were associated with CeD in *HLA-DQ2.5*-matched case-control groups. We reaffirm the association of the *BTN3A2* locus with CeD risk and identify *BTN2A1* and *BTN3A1* as putative novel CeD risk loci.

## 1. Introduction

### 1.1. Background to Coeliac Disease

Coeliac disease (CeD) is a T cell-mediated autoimmune enteropathy triggered by the consumption of gluten, a protein found in wheat, rye, and barley [1]. During active CeD, individuals with underlying genetic risk suffer from small intestinal inflammation after the consumption of dietary gluten [2]. This chronic inflammation causes villous atrophy that can lead to symptoms including abdominal pain, diarrhoea, malabsorption, and malnutrition [3]. Currently, the only treatment for CeD is eliminating gluten from the diet of patients with CeD predisposition [4].

The genetic background of CeD predisposition is still not fully understood, as only 50% of the genetic heritability has been attributed to specific loci [1]. The most well-established CeD risk loci are the human leukocyte antigen (*HLA)* complex [1,5,6,7,8,9,10]. The HLA-DQ2.5, HLA-DQ2.2, and HLA-DQ8 heterodimers are present in more than 80% of CeD patients [11,12,13,14,15]. In contrast, about 20–30% of healthy controls have the CeD-associated risk *HLA* genotypes [11,12,16]. These *HLA* genotypes were estimated to explain about 30–40% of the total CeD genetic heritability [17,18]. Although these *HLA* genotypes greatly contribute to CeD predisposition, non-*HLA* loci are increasingly becoming regions of interest in exploring the remaining 50% of CeD heritability. In order to further understand CeD susceptibility, genes involved in immunoregulatory pathways must be examined, such as the butyrophilin family of immunomodulators. Recent evidence has shown the butyrophilin family genes to be non-*HLA* CeD risk loci of interest [19,20,21].

### 1.2. The Emerging Role of the Butyrophilin Family of Genes and Their Role in Maintaining γδ T Cells

**Figure 1:**
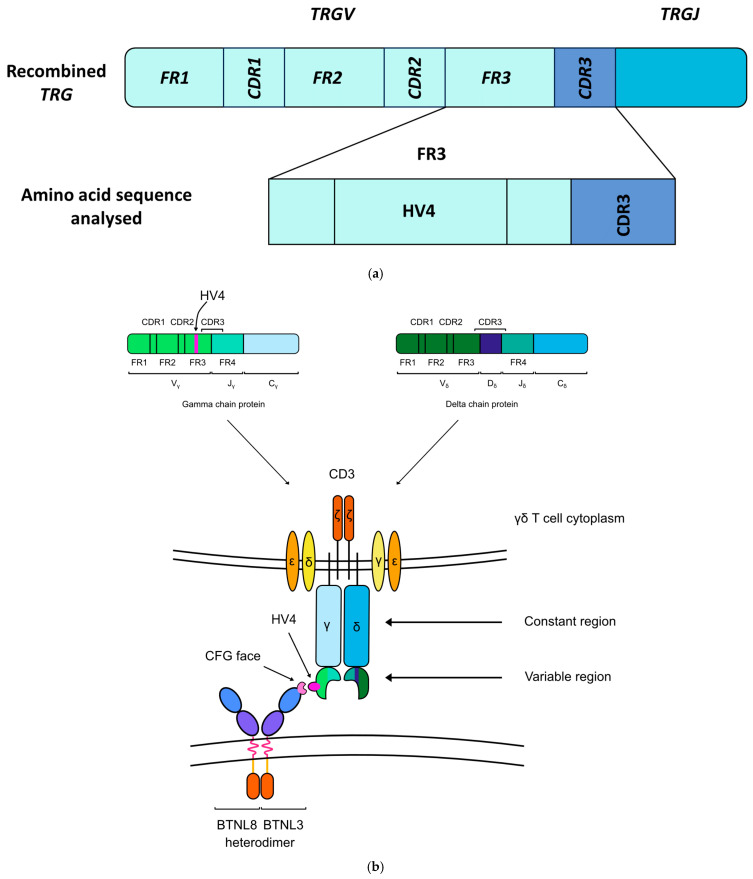
The germline-encoded HV4 loop of the T cell receptor (TCR) of Vγ4+ γδ IELs directly binds to BTNL3. (**a**) HV4 is located at amino acid positions 10–25 in the FR3 of the TRGV4 segment [31]. (**b**) The HV4 of Vγ4+ γδ T cells binds to the C, C”, F, and G canonical immunoglobulin-fold β-strands (CFG face) of the BTNL3 protein [29]. Abbreviations: CDR: complementarity-determining region; FR: framework region; HV4: hypervariable region 4; TRGJ: T cell receptor γ joining region; TRGV: T cell receptor γ variable region.

The butyrophilin proteins are a family of immunoglobulin-like cell surface receptors that have been shown to regulate both innate and adaptive immunity, including the activity of dendritic cells (DC), natural killer (NK) cells, αβ T cells, and γδ T cells [22,23,24,25,26]. Members of the butyrophilin family were found to maintain local γδ T cell compartments in the blood and epithelia of both mice and humans (Table 1) [27,28,29,30,31,32,33]. Hayday and Vantourout [34] hypothesised that butyrophilin proteins serve as a steady-state signal that maintains the local γδ T cell population in a quiescent or inactive state. In the duodenum, the BTNL3/BTNL8 heterodimers act as the ligand for Vγ4+/Vδ1+ γδ intraepithelial lymphocytes (IELs) (Figure 1) [21,27,28]. Specifically, the BTNL3/BTNL8 heterodimer binds the germline-encoded hypervariable region 4 (HV4) of T cell receptor gamma (TCR-γ), when the variable (V) gene segment encoding that TCR-γ is the *TRGV4* gene.

During active CeD, these γδ T cells, alongside CD4+ and CD8+ αβ IELs, are activated by dietary gluten [40]. Mayassi et al. [21] showed the loss of interaction between BTNL3/BTNL8 heterodimers and the duodenal Vγ4+ γδ T cells as a characteristic of active CeD in a study of 62 active CeD, 57 gluten-free diet (GFD)-treated CeD, and 99 control participants. During chronic inflammation induced by dietary gluten, the expression of the BTNL3/BTNL8 heterodimer was lost in the small intestine of patients with CeD predisposition. This was accompanied by the permanent loss of BTNL3/BTNL8-reactive Vγ4+/Vδ1+ γδ T cells. The chronic inflammation only subsided when patients followed a GFD. Although the BTNL3/BTNL8 expression recovered, the local γδ TCR repertoire was permanently reshaped: the innate-like Vγ4+/Vδ1+ γδ T cells and T cell receptor γ variable region 4 (*TRGV4)* gene transcripts were significantly decreased [21].

### 1.3. A Hypothesis for the Role of Butyrophilin Variation and γδ T Cells in CeD Risk

Recently, a common BTNL8*BTNL3 deletion copy number variant (CNV) was described by Aigner et al. [41] in a cohort of more than 4000 samples (Appendix A). The study reported that 58.4% of their 346 samples of European ancestry had at least one BTNL8*BTNL3 deletion allele (Table A1). This CNV has been shown to encode a BTNL8*3 fusion protein, which likely has an impaired ability to bind to the Vγ4Vδ1+ T cells in the small intestine [31]. As Mayassi et al. [21] observed a permanent shift in the duodenal γδ TCR repertoire when the interaction between the T cells and the BTNL3/BTNL8 heterodimer was disrupted, this fusion protein could predispose carriers to CeD.

Alongside *BTNL3* and *BTNL8*, *BTNL2* and *BTN3A1* were also implicated in CeD risk. Goudey et al. [19] have identified 14 SNPs associated with CeD, independent of the known CeD risk *HLA* loci, in a study of 763 CeD and 1420 control samples. One of the SNPs was located in the proximity of *BTNL2,* a gene harbouring among the highest density of GWAS hits in autoimmune and inflammatory diseases from the butyrophilin family [42,43,44,45,46,47]. Goudey et al. [19] showed that this SNP was marked as being an expression quantitative trait locus (eQTL) for the *BTNL2* gene in RegulomeDB, a database annotating the function of non-coding SNPs [48,49]. Furthermore, RegulomeDB also reported a high level of evidence for transcription factor binding for this eQTL [19].

In a separate paediatric study of 26 active CeD, 5 treated CeD, and 25 control subjects, *BTN3A1* expression was associated with active CeD in children [20]. The study examined the differential expression of more than 25 defence-related genes in the three subject groups, demonstrating the upregulation of *BTN3A1* mRNA and protein expression in the intestinal epithelial cells of children with active CeD. This is an intriguing finding, as BTN3A1 is required for the phosphoantigen (pAg)-induced activation of Vγ9Vδ2+ T cells in peripheral blood, a subset of γδ T cells not previously implicated in CeD. These two studies indicate that the full functions and roles of the butyrophilin family of proteins in immunomodulation remain to be explored.

These findings raise a previously unexplored question about CeD heritability. Do certain individuals co-inherit polymorphisms in their butyrophilin family genes and/or their *TRGV4* gene segments that predispose them to CeD? The objective of this study was to assess the association of butyrophilin gene-based burden with CeD risk using a 94-patient discovery cohort. The association of butyrophilin SNPs with CeD predisposition was validated via the UK Biobank’s genome-wide genotyping dataset of 25,192 participants. In this study, we show that 14 *BTN2A1*, 10 *BTN3A1,* and 13 *BTN3A2* SNPs are significantly associated with CeD status, while HV4 sequence variation was not associated with CeD risk.

## 2. Results

**Figure 2:**
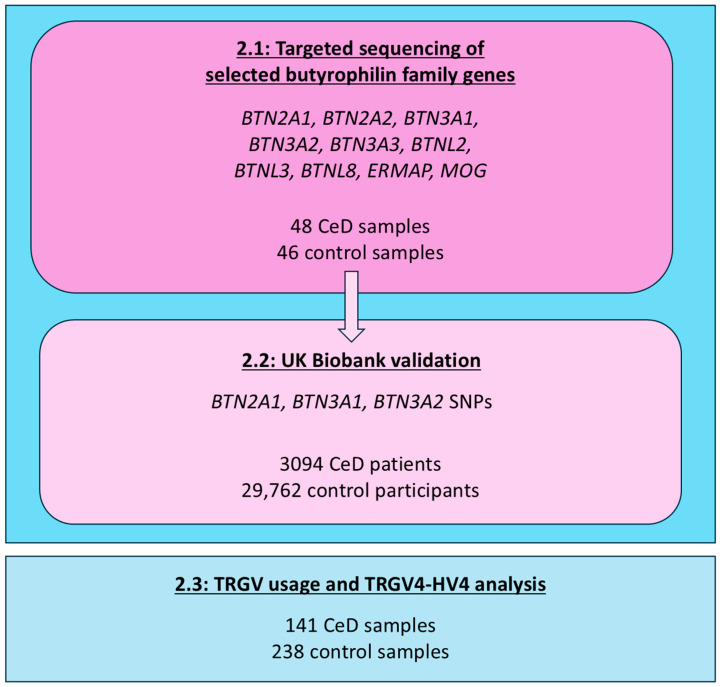
Workflow of the study on the association of the butyrophilin family loci and the TRGV4-HV4 sequences with CeD predisposition.

The impact of genetic variation in the butyrophilin family of genes and the HV4 sequence of duodenal γδ T cells on CeD predisposition was examined in three studies (Figure 2). First, 48 CeD and 46 control samples were subjected to targeted sequencing to capture SNPs in 10 butyrophilin family genes known to be expressed in small intestinal tissues and immune cells. The sequenced butyrophilin variance in CeD samples was burden tested via the control samples. Next, these results were validated, subjecting all available *BTN2A1*, *BTN3A1*, and *BTN3A2* SNPs to single-variant testing, in a cohort of 3094 CeD and 29,762 control participants from the UK Biobank genome-wide genotyping database. Finally, targeted sequencing of the TRGV4-HV4 sequence was undertaken in 141 CeD and 238 control samples, to investigate the association between TRGV4-HV4 variation and CeD risk.

### 2.1. BTN2A1 SNPs Were Significantly Associated with CeD Risk in a Study of 94 Samples

To investigate the association between butyrophilin genes and CeD risk, a cohort of 48 CeD and 46 control patients was examined for SNPs in 10 butyrophilin family genes, selected based on their gene expression profile in the duodenum, small intestines, and immune cells (Table A2 and Table A3) and their role in immunomodulation: *BTN2A1*, *BTN2A2*, *BTN3A1*, *BTN3A2*, *BTN3A3*, *BTNL2*, *BTNL3*, *BTNL8*, *ERMAP*, and *MOG*.

#### 2.1.1. Risk-Associated *HLA* Genotypes Were Significantly More Frequent in CeD Patients

First, by way of data quality control, the *HLA* genotypes of the samples were examined. In accordance with previous literature, 95.8% (46/48) of the CeD patients, compared with 54.3% (25/46) of the control group, had CeD risk-associated *HLA* genotypes (Fisher’s exact test, *p* = 5.5 × 10^−10^) (Table A7, Figure A6) [11,13,50].

#### 2.1.2. The BTNL8*BTNL3 Copy Number Variant Was Not Associated with CeD

Next, the *BTNL8-BTNL3* loci were examined for the presence of the deletion CNV. The presence of the CNV was determined using a surrogate SNP, the rs72494581 minor allele known to be associated with the presence of the deletion variant [51]. A total of 58.3% (28/48) of the CeD patients and 47.8% (22/46) of the control participants were found to possess at least one deletion variant (Table A8). Interestingly, 10.9% (5/46) of controls were homozygous for the BTNL8*BTNL3 deletion compared to only 4.2% (2/48) of CeD patients, but this did not reach statistical significance (Table A8, Figure A7, Fisher’s exact test, *p* = 0.2144).

#### 2.1.3. *BTN2A1* Gene Burden Was Significantly Higher in CeD Patients

To determine whether any of the butyrophilin family variants were associated with CeD risk, gene-based burden testing, using the TRAPD program [52], was carried out to burden test the non-synonymous coding variants identified in the CeD patients against the variants in the control samples.

The analysis was carried out on qualifying variants at sites where more than 90% of samples had a read depth coverage of >10. Of the 108 and 58 non-synonymous coding variants discovered in the CeD and control samples, respectively, only 5 bi-allelic SNPs shared by both the CeD and control groups qualified for burden testing (Table 2, Table A9, Table A10 and Table A11). Only *BTN2A1* variants were significantly associated with CeD risk gene burden in both the dominant (adjusted *p* = 1.46 × 10^−5^) and the recessive (adjusted *p* = 3.70 × 10^−8^) models, indicating that the presence of a single qualifying *BTN2A1* SNP significantly increased CeD risk (Table 2a,b). *BTN2A1* variants were more frequent in CeD patients, as 45.8% (22/48) of CeD participants had at least one qualifying *BTN2A1* variant compared to 10.9% (5/46) of controls (Table 2a,b). To summarise, the gene burden analysis of butyrophilin genes in CeD patients compared with controls showed a significant association between *BTN2A1* gene burden and CeD risk.

Although these results were promising, due to the *BTN2A1* gene being part of the extended MHC region and its close proximity (~4 Mb) to the classical MHC region (6p21.3), we could not exclude the possibility that this significant association could be secondary to the risk-associated *HLA* genotypes of the CeD patients [54,55]. Therefore, these results were validated in the 500,000 genome-wide genotyping dataset of the UK Biobank, by single-variant testing of *BTN3A1*, *BTN3A2*, *BTN2A1*, *BTNL3*, and *BTNL8* SNPs.

### 2.2. BTN3A1, BTN3A2, and BTN2A1 Genes Were Significantly Associated with CeD in HLA-DQ2.5-Matched Participants of the UK Biobank Database

The UK Biobank dataset was used to validate the association between *BTN2A1* and CeD risk and to investigate the association between CeD and butyrophilin SNPs in potentially CeD-relevant genes. After removing participants with missing HLA imputation or genotype data, the final cohort consisted of 3094 CeD patients and 29,762 control participants (Appendix H).

#### 2.2.1. Risk-Associated *HLA* Genotypes Were Significantly More Frequent in CeD Patients of the UK Biobank

First, as a means of quality control for CeD diagnosis, the *HLA* genotypes of the CeD and control participants of the UK Biobank were examined. The majority of participants selected from the 500,000 genome-wide genotyping dataset had CeD risk *HLA* genotypes regardless of their CeD status (Table A13, Figure A12). Risk *HLA* genotypes were found in 92.4% (2860/3094) of CeD patients and 57.6% (17,144/29,762) of controls. In both control and CeD participants, *HLA-DQ2.5* was the most frequent *HLA* genotype at 21.6% (6416/29,762) and 53.4% (1652/3094), respectively. Interestingly, *HLA-DQ8* was the second most frequent risk genotype in controls at 14.1% (4203/29,762). Meanwhile, individuals heterozygous for HLA-DQ2.5/HLA-DQ8 were the second most frequent in the CeD group, with 19.6% (606/3094) of participants possessing that risk *HLA* genotype.

To compare the proportion of CeD risk-associated *HLA* genotypes in CeD and control participants in the 500,000 genome-wide genotyping dataset, a chi-square test of independence was used. Similar to the results from the targeted butyrophilin sequencing dataset, the CeD participants had significantly higher proportions of CeD risk *HLA* genotypes compared with controls (X-squared = 4062.5, df = 6, *p* < 2.2 × 10^−16^).

Indeed, when the association between the CeD risk *HLA* genotypes and CeD status was investigated using a binomial regression model in the UK Biobank dataset, the association between the risk *HLA* genotypes and CeD status was confirmed. Interestingly, in the regression analysis, all risk *HLA* genotypes were significantly associated with CeD (adjusted *p* ≤ 5.13 × 10^−4^, Table A14) except the *HLA-DQ8* genotype (adjusted *p* = 0.125).

#### 2.2.2. *BTN2A1*, *BTN3A1*, and *BTN3A2* SNPs Were Significantly Associated with CeD Status in the UK Biobank

Single-variant analyses were carried out to test the association between CeD status and SNPs from the *BTN3A1*, *BTN3A2*, *BTNL3*, and *BTNL8* genes in the UK Biobank [56]. Due to the genotyping array used by the UK Biobank, the genetic information of only a limited number of SNPs from each gene was available. A total of 101 butyrophilin SNPs were individually tested for association with CeD status in the UK Biobank (Table 3). As the *HLA* loci were significantly associated with CeD risk [1], and the *BTN3A1* and *BTN3A2* loci are in close proximity [22,24], the CeD risk *HLA* genotypes were also taken into account for the single-variant analyses by including the risk *HLA* genotypes in the binomial models and analysing the association between butyrophilin SNPs and CeD status in HLA-matched case-control groups as well. The genetic associations were tested by building binomial regression models, where the association between each variable and CeD status was examined.

A total of 37 SNPs were significantly associated with CeD status in the UK Biobank: 14 *BTN2A1*, 10 *BTN3A1*, and 13 *BTN3A2* SNPs (adjusted *p*-value ≤ 0.05, Table 4, Table A15 and Table A16). All 37 SNPs were in Hardy–Weinberg equilibrium in the control cohort (Table A17). Most of the significant SNPs were non-coding, with 25 of the 37 SNPs being located in intronic regions. Only one *BTN3A1* (rs41266839) and three *BTN2A1* (rs13195509, rs3734542, and rs3734543) SNPs were missense variants, and one *BTN2A1* (rs13195402) SNP encoded a STOP codon. Of the 37 SNPs, the reference alleles of 30 SNPs were associated with a decreased CeD risk. No *BTNL3* nor *BTNL8* SNPs were significant in predicting CeD status in the UK Biobank dataset, after Bonferroni correction.

#### 2.2.3. Twenty Butyrophilin SNPs from the UK Biobank Remained Significantly Associated with CeD Status When the Participants’ Risk *HLA* Genotypes Were Taken into Account

To investigate whether the butyrophilin SNPs in the UK Biobank remained significantly associated with CeD status when taking the *HLA* loci into account, a second set of binomial regression models was produced. Single-variant models were built for each of the 101 SNPs of interest, which included the risk *HLA* genotypes of the UK Biobank participants as an additional predictor variable (Table A18). Only 7 *BTN2A1*, 2 *BTN3A1*, and 11 *BTN3A2* SNPs remained significantly associated with CeD status after applying Bonferroni correction (adjusted *p* ≤ 0.05, Table 5). All of the significant SNPs were in Hardy–Weinberg equilibrium in the control cohort (Table A19). Similar to the previous model, the majority of the significant SNPs were non-coding, with the exception of a STOP gained SNP (rs13195402) and three missense SNPs (rs13195509, rs3734542, and rs3734543) in the *BTN2A1* gene. Out of the 17 non-coding SNPs, 11 SNPs were located in intronic regions. The reference alleles for all 20 SNPs were associated with a decreased CeD risk, meaning that the alternate alleles were more frequent in CeD patients. As the *HLA* loci were taken into account, these SNPs are likely to be real associations with CeD status, instead of being caused by linkage disequilibrium (LD) due to the proximity of the *BTN* and *HLA* loci on chromosome 6.

#### 2.2.4. Twenty-One Butyrophilin SNPs Were Significantly Associated with CeD Status in *HLA-DQ2.5*-Matched Case-Control Groups of UK Biobank Participants

The final set of analyses was carried out to investigate whether the butyrophilin SNPs were significantly associated with CeD status in all of the CeD risk *HLA* genotype patients. Therefore, the UK Biobank participants were separated into risk *HLA*-matched CeD and control groups (Table 6). All 101 butyrophilin SNPs were single-variant tested for their association with CeD status in the *HLA*-matched groups.

*HLA-DQ2.5* was the most common risk *HLA* genotype in CeD patients, both in the UK Biobank as well as in previous studies [11,12]. A significant association between butyrophilin SNPs and CeD status was only present in the *HLA-DQ2.5*-matched UK Biobank participants. The *BTN2A1*, *BTN3A1*, and *BTN3A2* SNPs significantly associated with CeD status in the *HLA* single-variant testing models remained significant in the *HLA-DQ2.5*-matched tests as well (Table 7, Table A20 and Table A21). Interestingly, the allele frequency of all significantly associated SNPs significantly differed from the Hardy–Weinberg equilibrium in the control group (Table A22). Additionally, rs7773938, an intronic *BTN2A1* SNP, is a novel SNP that was only significantly associated with CeD status in UK Biobank participants with the *HLA-DQ2.5* genotype. The reference alleles of all 21 significant SNPs were more frequent in controls compared to CeD individuals, meaning that having the alternate allele at these loci significantly increases an individual’s CeD risk. These results imply that butyrophilin SNPs could only explain additional CeD risk in HLA-DQ2.5-matched individuals of the UK Biobank. As the presence of these reference alleles remained significantly associated with decreased CeD risk even after *HLA*-matching, the association with these SNPs was not likely to be caused by LD to the *HLA* loci. Therefore, the 21 butyrophilin SNPs identified were significantly associated with CeD status and contributed to further CeD risk in UK Biobank participants possessing the *HLA-DQ2.5* genotype.

### 2.3. HV4 Variation Was Not Significantly Associated with CeD Risk in a Study of 379 Samples

#### 2.3.1. TRGV Usage Was Not Significantly Different Between CeD and Control Samples

Previous evidence by our group showed that the γδ T cell repertoire is permanently altered in the duodenum of CeD patients [57]. Mayassi et al. [21] also showed that the BTNL3-reactive duodenal Vγ4+ γδ T cells are lost after active CeD, and the local γδ TCR repertoire is permanently reconfigured.

**Figure 3:**
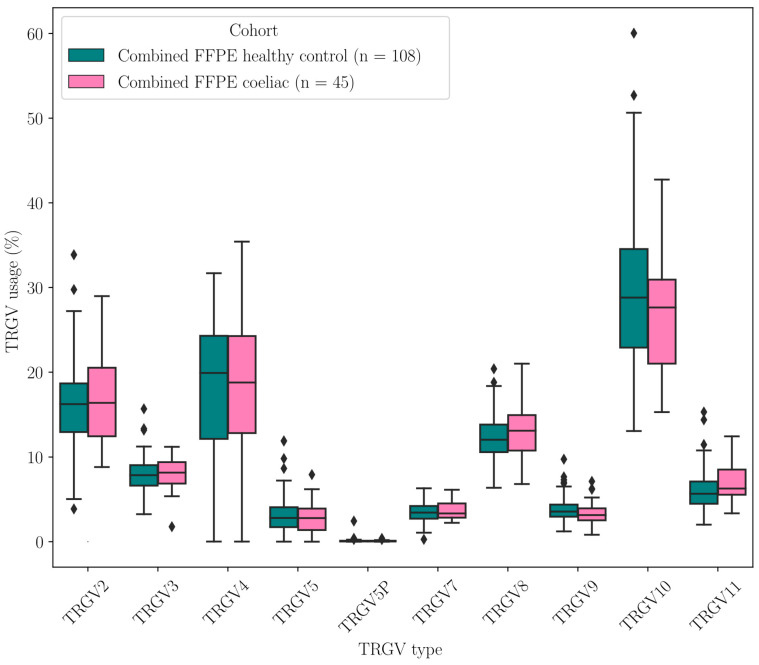
There were no significant differences in the TRGV usage of CeD (n = 45) and healthy control (n = 108) duodenal samples.

First, we investigated TRGV usage in the duodenal TRG repertoires of 108 healthy controls and 45 CeD patients (Table 8, Figure A13). The *TRGV10*, *TRGV4*, and *TRGV2* variable (V) gene segments were the most frequent in this dataset. We focused on the TRGV4 segment usage, which is capable of binding the BTNL3/BTNL8 heterodimer. The mean TRGV4 segment usage did not differ between CeD (18.50% of the TRG repertoire) and healthy control samples (18.06%) (Figure 3, Table A23).

#### 2.3.2. HV4 Sequence Variation Was Not Significantly Associated with CeD Risk

Next, the TRGV4-HV4 amino acid sequences were examined in 141 CeD and 238 healthy control samples (Table 8). As demonstrated by Melandri et al. [29] and Willcox et al. [31], only HV4 loops with the wild-type (reference) KYDTYGSTRKNLRMILR amino acid sequence could directly bind BTNL3. Substitutions in the amino acids underlined (KY**D**TY**G**STRKNLRMILR) were found to disrupt this direct binding between BTNL3 and Vγ4+ T cells, while substitutions in the following underlined amino acids (KYDTYGSTR**K**NLR**M**ILR) only caused a marginal reduction in binding [31]. As the HV4 is germline-encoded and does not undergo recombination, we hypothesised that variations in the germline-encoded TRGV4-HV4 amino acid sequence could alter the binding of the Vγ4+ γδ T cells to BTNL3 protein in the duodenum, predisposing to CeD.

Seven unique HV4 amino acid sequences were identified in the dataset (Table 9a,b). The reference HV4 sequence KYDTYGSTRKNLRMILR capable of binding the BTNL3 protein was the most frequent in both the healthy control (95.8%, 228/238) and CeD (97.9%, 138/141) samples. Approximately 84.9% (202/238) of healthy control samples and 82.3% (116/141) of CeD were homozygous for the WT HV4 sequence. There were no significant differences in the HV4 amino acid sequence distribution between CeD and healthy control samples (Fisher’s exact test, *p* = 0.26, Figure A14, Table A24). Thus, neither TRGV usage nor HV4 amino acid sequence variation could explain CeD risk in a dataset of 379 samples.

## 3. Discussion

Around 30% of genetic heritability for CeD can be explained by the *HLA* risk genotypes *HLA-DQ2.5*, *HLA-DQ8*, and *HLA-DQ2.2*, which were first connected to CeD in 1972 [17,18,58,59]. However, an estimated 50% of genetic heritability remains unexplored [60]. Recently, the butyrophilin family of genes were proposed as non-*HLA* CeD risk loci [19,20,21]. These genes encode transmembrane proteins that were implicated in regulating the activity of innate and adaptive immune cells, alongside maintaining characteristic epithelial γδ T cell populations in mice and humans [22,24]. Prior to this study, four genes were associated with CeD: *BTN3A1*, *BTNL2*, *BTNL3*, and *BTNL8* [19,20,21].

Burden testing the non-synonymous coding butyrophilin SNP data of 46 healthy control and 49 CeD samples showed the *BTN2A1* gene burden to be significantly higher in CeD patients in both the dominant (adjusted *p* = 1.46 × 10^−5^) and the recessive models (adjusted *p* = 3.70 × 10^−8^). CNV analysis of the *BTNL8-BTNL3* region in these samples did not show a significant association with CeD risk.

The significant association between *BTN2A1* SNPs and CeD predisposition was validated using the UK Biobank 500,000 genome-wide genotyping dataset. Fourteen *BTN2A1*, 10 *BTN3A1,* and 13 *BTN3A2* SNPs were significantly associated with CeD (adjusted *p* ≤ 0.05), the majority of which were non-coding variants. When the risk-associated *HLA* genotypes of these participants were taken into account, only 7 *BTN2A1*, 2 *BTN3A1,* and 11 *BTN3A2* SNPs remained significant (adjusted *p* ≤ 0.05), showing *HLA*-independent associations with CeD risk. Finally, butyrophilin SNPs were single-variant tested in CeD risk *HLA*-matched groups. The 20 SNPs above, alongside a novel intronic *BTN2A1* SNP, were significant in predicting CeD status in 1652 CeD and 6416 control participants with the *HLA-DQ2.5* genotype (adjusted *p* ≤ 0.05).

We thus identified *BTN2A1* and *BTN3A2* as novel CeD risk loci and corroborated *BTN3A1* as a CeD risk locus. The association between *BTN3A1* and CeD is in accordance with evidence shown by Pietz et al. [20], who hypothesised that the pAg presentation by intestinal epithelial cells in active CeD may contribute to IFN-γ production and T cell proliferation. Indeed, all three of these butyrophilin genes are involved in the pAg-mediated, innate-like activation of peripheral blood γδ T cells [37,38,61,62]. Interestingly, the majority of the UK Biobank SNPs significantly associated with CeD predisposition were outside coding regions. Of note, UK Biobank validation indicated that only non-coding *BTN3A1* and *BTN3A2* SNPs were significantly associated with CeD risk. These results could provide an explanation for these genes not having significantly increased gene burden in CeD patients, as the burden testing only considered coding variants.

**Figure 4:**
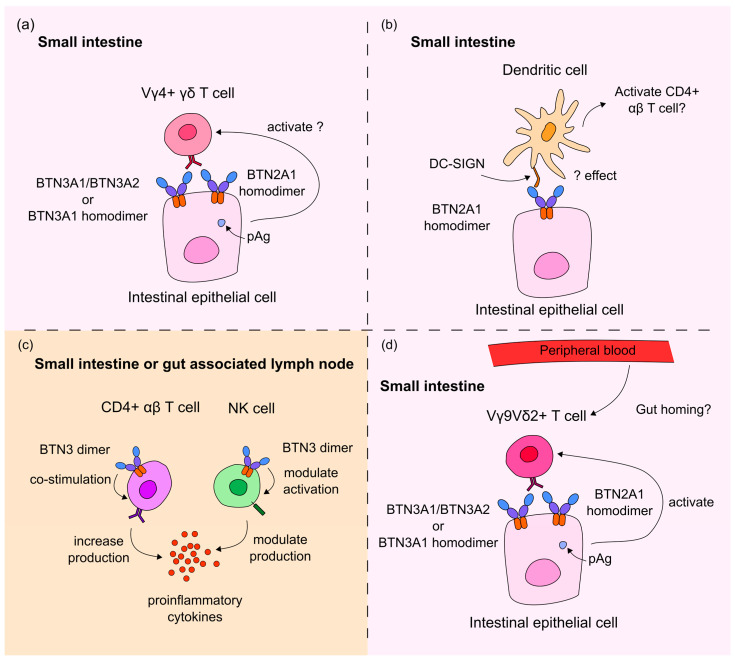
BTN2A1, BTN3A1, and BTN3A2 may be involved in CeD pathogenesis by modulating T cell and innate immune cell activity. *BTN2A1* gene burden was significantly higher in CeD patients in a cohort of 94 samples. Meanwhile, *BTN2A1*, *BTN3A1,* and *BTN3A2* SNPs were significantly associated with CeD status in the UK Biobank database. Based on our results and evidence on the immunomodulatory role of butyrophilins on innate and adaptive immune cells, butyrophilins could contribute to CeD pathogenesis in multiple potential manners [25,26,37,38,61]: (**a**) via the novel, hypothesised pAg-dependent activation of Vγ4+ γδ T cells; (**b**) via the interaction of BTN2A1 with dendritic cells through the DC-SIGN receptor on the DC cell surface; (**c**) by increasing the co-stimulation and IFN-γ production of CD4+ αβ T cells, or by modulating the activity and IFN-γ production of NK cells depending on whether BTN3A1 or BTN3A2 is expressed predominantly on the surface of the NK cell; or (**d**) via the pAg-dependent activation of potentially gut-homing Vγ9Vδ2+ γδ T cells in the small intestine.

Due to the association between butyrophilin family members and CeD status, and the involvement of butyrophilin heterodimers in shaping γδ T cell repertoires via binding to Vγ4+ γδ T cells [21,27,28,31], we investigated the likely effects of polymorphisms in the TCR γ V segment, TRGV4, on the interaction between Vγ4+ γδ T cells and the BTNL3/BTNL8 heterodimer, but we failed to find any significant association between the TRGV4-HV4 amino acid sequences and CeD risk, which may suggest that the interaction between Vγ4+ γδ T cells and the BTNL3/BTNL8 heterodimer is not a primary event in determining whether or not CeD develops. However, these results could be due to both the coding regions of butyrophilin genes and the HV4 amino acid sequence being conserved via stabilising selection. This could be due to the interaction between butyrophilins and γδ T cells, including *BTN3A1* and *BTN3A2* PAg-dependent activation of peripheral blood Vγ9+ T cells, and HV4-BTNL3 interaction, which serves as the maintenance signal for the Vγ4+ γδ T cells in the duodenum [21]. Taking these results together, we provide a new hypothesis for the role of butyrophilins in CeD (Figure 4).

Firstly, these results could imply that BTN2A1 and BTN3A2 act on duodenal Vγ4+ γδ T cells, as well as on peripheral blood Vγ9Vδ2+ γδ T cells, perhaps mediating their pAg-dependent activation (Figure 4a). This hypothesis could explain why the BTNL8*BTNL3 deletion variant, which encodes a BTNL8*3 fusion protein but no full-length BTNL3 or BTNL8 proteins, was not significantly associated with CeD risk in the cohort of 94 samples. Participants who are homozygous for the deletion can only express the truncated BTNL8*3 fusion protein, which lacks the BTNL3-IgV extracellular domain required for maintaining the duodenal TCR of Vγ4+ γδ T cells, which we hypothesised could increase CeD risk [21,29,31,41]. If BTN2A1, BTN3A1, or BTN3A2 could provide a survival signal to the Vγ4Vδ1+ IELs in the healthy small intestine, this could explain why controls could be homozygous for the BTNL3/BTNL8 deletion variant without having CeD.

Secondly, BTN2A1 variants may predispose patients to CeD, via BTN2A1’s role as a ligand for DC-SIGN on DCs, which are important in CD pathogenesis in presenting gluten antigens to CD4+ αβ T cells [24,25]. Thus, BTN2A1 might regulate the autoimmune response in CeD indirectly via DC activity (Figure 4b). Additionally, previous evidence has shown that BTN3 proteins can provide co-stimulatory signals to αβ T cells, increasing their production of interferon-γ (IFN-γ), a proinflammatory cytokine [26]. This same study showed the dual effect of butyrophilins on NK cell activity: BTN3A1 upregulated, while BTN3A2 downregulated IFN-γ production and NK cell activation (Figure 4c).

Thirdly, peripheral blood Vγ9Vδ2+ T cells might undergo BTN2A1-mediated PAg-dependent activation in CeD (Figure 4d), either being recruited to infiltrate the small intestine from the peripheral blood or contributing to CeD pathogenesis in an as yet undetermined way. Interestingly, in the analysis of our cohort of 108 healthy control and 45 CeD duodenal samples, only 3–4% of γδ T cells were Vγ9+ T cells (Figure 3, Table 8). There were no significant differences in the proportion of Vγ9+ T cells in CeD and healthy controls (adjusted *p* = 0.728), a finding which may argue against a key role for Vγ9+ T cells in CeD.

In conclusion, the butyrophilin family of genes are promising immunomodulators involved in connecting the adaptive and innate immunity [24]. Our results provide evidence that the butyrophilin genes *BTN2A1*, *BTN3A1*, and *BTN3A2* may be putative CeD risk loci. Due to their important roles in the maintenance, activation, and regulation of γδ T cells, the butyrophilins may be involved in the pathogenesis of other autoimmune and inflammatory disorders. Our work provides a clear rationale for further research into the role of the butyrophilin family of genes in CeD.

## 4. Materials and Methods

### 4.1. Participant Selection Criteria

All patient samples used for sequencing were obtained with full ethical approval (IRAS project ID: 162057, REC reference: 04/Q1604/21, PI: Prof. E. Soilleux).

CeD patient samples were selected using hospital records, while control samples were selected to exclude suspected CeD patients.

Control exclusion criteria:

- Has CeD diagnosis;
- Malabsorption;
- Anaemia;
- Lymphocytosis;
- On a GFD;
- Diarrhoea.

#### 4.1.1. Participant Selection for the Butyrophilin Family Gene Sequencing

A total of 48 CeD samples (40 blood, 8 formalin fixed, paraffin-embedded (FFPE) duodenal biopsies) and 46 control samples (38 blood, 8 FFPE duodenal biopsies) were obtained from Cambridge Haematopathology and Oncology Diagnostic Service or Cambridge University Hospitals NHS Foundation Trust Department of Haematology (blood samples) and the Human Research Tissue Bank of Cambridge University Hospitals NHS Foundation Trust (FFPE biopsies).

#### 4.1.2. Validation Cohort Participant Selection from the UK Biobank for Single-Variant Analysis

CeD patients and controls were selected from the anonymised UK Biobank online database using the Cohort Browser program on the online Research Analysis Platform (RAP, https://ukbiobank.dnanexus.com/, application ID: 18532, accessed on 23 May 2022). Participants’ sociodemographic, lifestyle, hospital record information, HLA imputation, and genome-wide genotyping data were available from the UK Biobank online resource centre (https://biobank.ndph.ox.ac.uk/, accessed on 23 May 2022).

Control and CeD participants were selected based on their responses to the CeD online questionnaire (data-field 21068, https://biobank.ctsu.ox.ac.uk/crystal/field.cgi?id=21086, accessed on 23 May 2022), the dietary web questionnaire (data-field 20086, https://biobank.ctsu.ox.ac.uk/crystal/field.cgi?id=20086, accessed on 23 May 2022), their hospital inpatient record (category 2000, https://biobank.ctsu.ox.ac.uk/crystal/label.cgi?id=2000, accessed on 23 May 2022), and their death record (category 100093, https://biobank.ctsu.ox.ac.uk/crystal/label.cgi?id=100093, accessed on 23 May 2022). All participant clinical data were classified using the World Health Organisation’s International Classification of Disease (ICD) system [63]. Most of the hospital inpatient data were coded in ICD-10, but some pre-1997 data collected in Scotland used ICD-9 (https://biobank.ndph.ox.ac.uk/ukb/refer.cgi?id=138483, accessed on 23 May 2022).

Control exclusion criteria were the same as for the blood and biopsy cohort, with the CeD online questionnaire, hospital inpatient record, or death record serving as evidence of a CeD diagnosis.

Coeliac disease inclusion criteria included either of the following:

- Hospital diagnosis record includes coeliac disease: ICD9 (5790), ICD10 (K90.0);
- Cause of death includes coeliac disease: ICD10 (K90.0).

After removing individuals with missing data, the finalised UK Biobank cohort consisted of 3094 CeD patients and 29,762 control participants.

#### 4.1.3. Samples Selected for the HV4 Analysis

The sequencing data from three different datasets were used that were selected using the same criteria. A total of 141 CeD and 238 healthy control tissue samples were selected for the HV4 analysis (Table 8).

### 4.2. Analysis of Butyrophilin Family Variation in the Targeted Sequencing Cohort

#### 4.2.1. Sequencing of *HLA* Loci and Selected Butyrophilin Family Genes by Hybridisation Capture

The expression profiles of the 15 butyrophilin family members outlined by Rhodes et al. [24] were examined in the Human Protein Atlas (HPA, accessed on 27 October 2020) for protein (or, where protein was unavailable, mRNA) expression in the duodenum, small intestine, rectum, and colon (Appendix B, Table A2), as well as mRNA expression in T cells, DCs, NK cells, macrophages, regulatory T cells, and γδ T cells (Table A3) [64]. *BTN2A1*, *BTN2A2*, *BTN3A1*, *BTN3A2*, *BTN3A3*, *BTNL2*, *BTNL3*, *BTNL8*, *ERMAP*, and *MOG* were selected.

**Figure A1:**
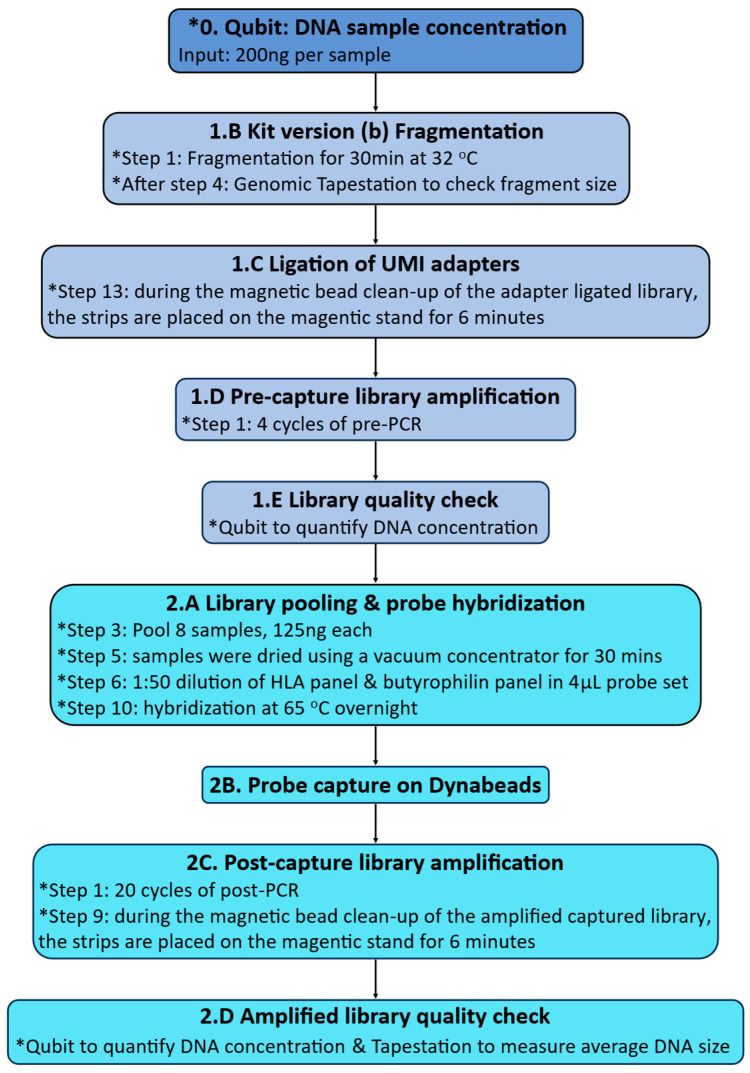
The Nonacus Cell3 capture hybridisation capture method was modified for the HLA and butyrophilin sequencing panel. All modifications to the manufacturer’s protocol were noted with *. The HLA probes were provided by Nonacus Ltd.

The Genome Reference Consortium Human Build 38 patch release 12 (GRCh38.p12) genomic position of the 10 butyrophilin genes of interest was determined using the NCBI database [65], the regions of interest were uploaded to the Nonacus Ltd. probe design platform (panel id: 890, Table A4) [66], and 2× tiling probes were designed maximising coverage of the target regions, while avoiding under or over sequencing any regions [67,68]. *HLA* hybridisation probes were designed and provided by Nonacus Ltd. Hybridisation capture was performed using the Nonacus Cell3 Target Hybridisation & Capture Kit (Nonacus) version (b) protocol (Figure A1, Appendix B.2 and Appendix B.3). Captured libraries were sequenced using the Illumina MiSeq system. Sequencing data obtained are available at https://zenodo.org/records/15203243 (accessed on 12 April 2025).

#### 4.2.2. Germline Short-Variant Discovery and *HLA* Genotyping

The quality of the sequencing files was assessed using the default FastQC v0.11.9 settings, and the Illumina adapters were removed using Trimmomatic v0.39 [69,70].

The variant call pipeline was built by adapting the GATK best practices for germline short-variant discovery [71], the analysis pipelines of Zhao et al. [72], the Du group [73,74], and Matthews [75] (Appendix C). The code for the pipeline calling SNPs from the raw, unmapped FASTQ sequencing files is available at https://gitlab.developers.cam.ac.uk/path/soilleux/soilleux-group/ced_butyrophilin_phd/-/tree/dropbox/nonacus_miseq_analysis/variant_call (accessed on 19 March 2025).

*HLA* genotypes were determined from the sequencing data using HLA-HD version 1.7.0 [76], and the CeD risk-associated *HLA* genotypes (Section 4.3.1) were called from the alleles. The code for the risk *HLA* genotyping is available at https://gitlab.developers.cam.ac.uk/path/soilleux/soilleux-group/ced_butyrophilin_phd/-/tree/dropbox/nonacus_miseq_analysis/hla_typing (accessed on 24 September 2024).

#### 4.2.3. Copy Number Variation (CNV) Analysis of the *BTNL8-BTNL3* Loci

The presence of the 56 kb deletion variant in the *BTNL8-BTNL3* loci (chr5:180948027–181003596, GRCh38) was analysed by using a surrogate SNP, the *T > C* rs72494581 (chr5:181003797, GRCh38) *BTNL3* intronic SNP, which is associated with the CNV (Table A5) [51]. Fisher’s exact test was performed to investigate differences in *BTNL8-BTNL3* CNV between cohorts.

#### 4.2.4. Burden Testing Analysis

**Figure A4:**
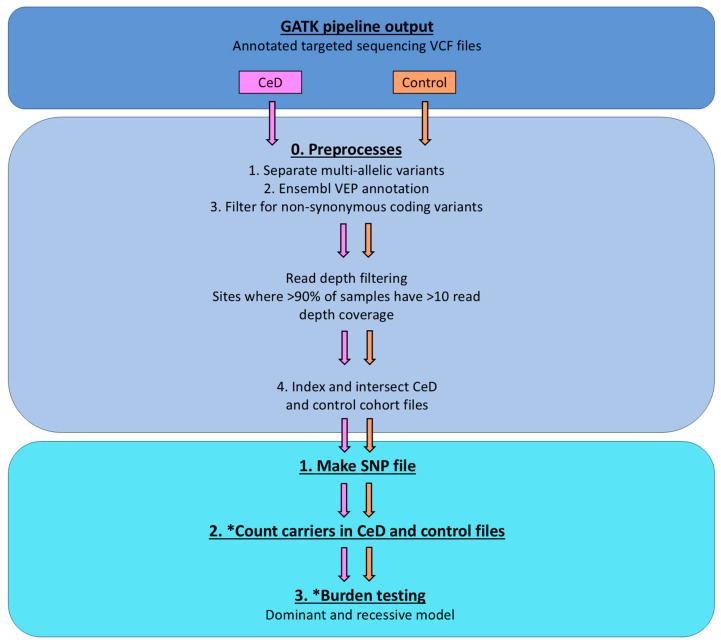
The variants in the CeD cohort (n = 48) were burden tested via the controls (n = 46) using the TRAPD program. Test Rare vAriants with Public Data (TRAPD) was used to burden test the variants in the hybridisation capture CeD cohort (n = 48) against the control cohort (n = 46) [52]. The annotated CeD and control files from the GATK pipeline were preprocessed as recommended by the manual [53]. The variants were burden tested after read depth filtering. Steps in which the code was modified are marked with *.

The TRAPD program was used for burden testing the variants found in selected butyrophilin genes in samples of the targeted sequencing cohort, as described in Appendix D.2 (Figure A4) [52,53,77,78].

The variants in the CeD and control groups were burden tested using both the recessive and the dominant models.

The burden testing analysis codes are available at https://gitlab.developers.cam.ac.uk/path/soilleux/soilleux-group/ced_butyrophilin_phd/-/tree/dropbox/nonacus_miseq_analysis/burden_testing/Code (accessed on 25 September 2024).

### 4.3. Single-Variant Testing of Butyrophilin Family Variance in the UK Biobank Database

#### 4.3.1. CeD Risk-Associated *HLA* Genotyping in the UK Biobank Cohort

HLA genotyping was performed using the HLA imputation values of the UK Biobank 500,000 genome-wide genotyping cohort (Appendix E.1.), to identify the following CeD risk-associated alleles: HLA-DQA1*02:01 with HLA-DQB1*02:02 (making up the HLA-DQ2.2 heterodimer in the DR2-DQ2 haplotype), HLA-DQA1*05:01 with HLA-DQB1*02:01 (making up the HLA-DQ2.5 heterodimer in the *DR3-DQ2* and *DR5-DQ7/DR7-DQ2* haplotypes), and HLA-DQA1*03:01 with HLA-DQA1*03:02 (making up the HLA-DQ8 heterodimer in the DR4-DQ8 haplotype).

#### 4.3.2. Single-Variant Testing Using Binomial Regression Models

The single-variant testing model was built into R version 4.2.1 by adapting the UK Biobank analysis of Yu et al. [79]. The code for investigating the association between butyrophilin family SNPs and CeD risk in the UK Biobank is available at https://gitlab.developers.cam.ac.uk/path/soilleux/soilleux-group/ced_butyrophilin_phd/-/tree/dropbox/ukbiobank_butyrophilin_snp/Butyrophilin_SNP_analysis?ref_type=heads (accessed on 13 November 2024).

The UK Biobank individual SNP data were annotated using the reference SNP cluster IDs (rsIDs) from the SNP database (dbSNP) and the reference allele for these SNPs from the Genome Reference Consortium Human Build 37 (GRCh37) [56,65,80,81]. Further methodological information can be found in Appendix E.2.

### 4.4. Analysis of TRGV Usage and HV4 Variation in CeD and Control Samples

#### 4.4.1. Processing Samples and TCR Sequencing

**Figure A5:**
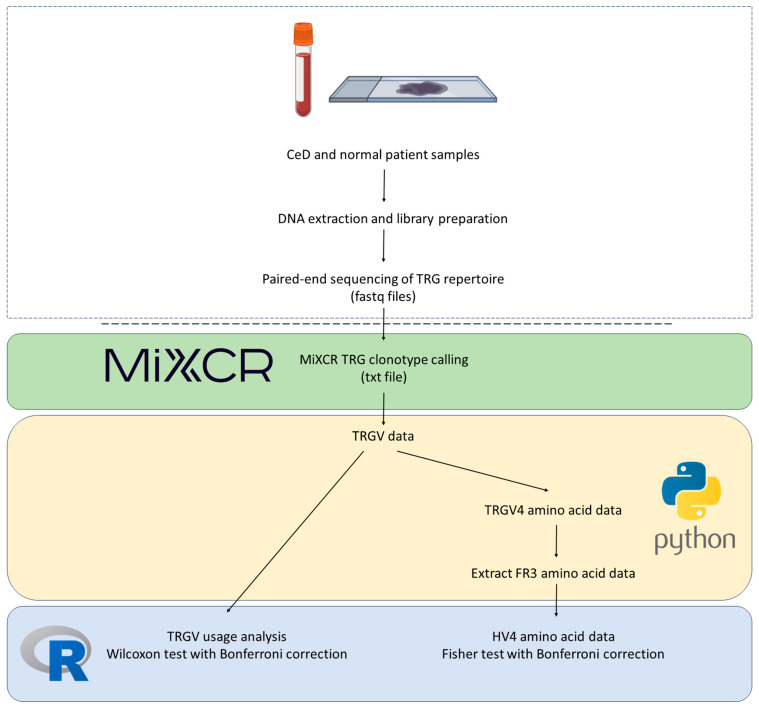
CeD and healthy control patient TRG sequencing data from three cohorts were used to analyse differences in TRGV usage and germline HV4 sequences.

**Figure A6:**
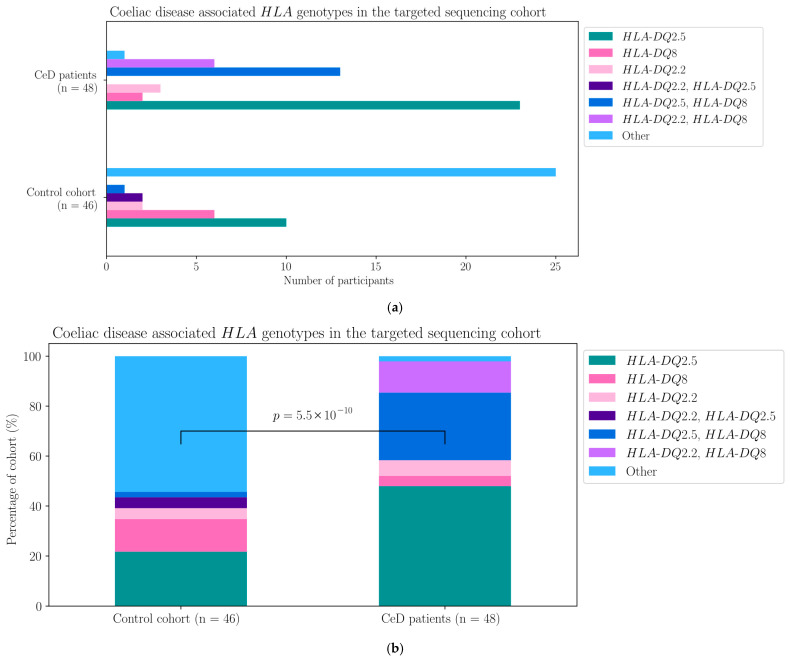
CeD patients (n = 46) had significantly higher proportions of CeD risk *HLA* genotypes compared to controls (n = 48). The (**a**) number and (**b**) percentage of individuals with CeD risk-associated *HLA* genotypes were significantly higher in CeD patients compared to controls in this dataset (Fisher’s exact test, *p* = 5.5 × 10^−10^). The *HLA* genotypes for participants were called using HLA-HD [95].

**Figure A7:**
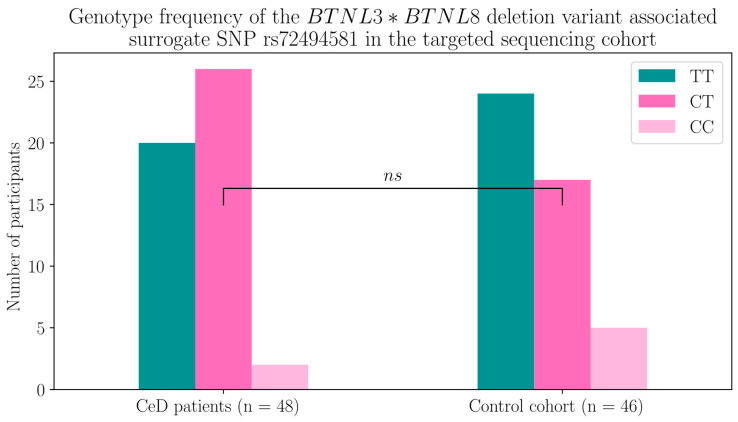
There were no significant differences in the frequency of the BTNL8*BTNL3 copy number variants between CeD patients (n = 48) and controls (n = 46). The BTNL8*BTNL3 deletion variant encodes a truncated BTNL8-BTNL3 fusion protein [41]. Dart et al. [51] identified rs72494581 in the intronic region of *BTNL3*, which serves as a surrogate SNP, and the alleles are associated with the BTNL8*BTNL3 copy number variants. The major allele, the *T* allele, is associated with the full-length *BTNL3* and *BTNL8* genes, while the minor allele, the *C* allele, is associated with the BTNL8*BTNL3 deletion. Fisher’s exact testing showed that there were no significant differences (adjusted *p* = 0.2144) in the frequency of the BTNL8*BTNL3 deletion variant-associated rs72494581 genotypes in the CeD and control groups.

**Figure A12:**
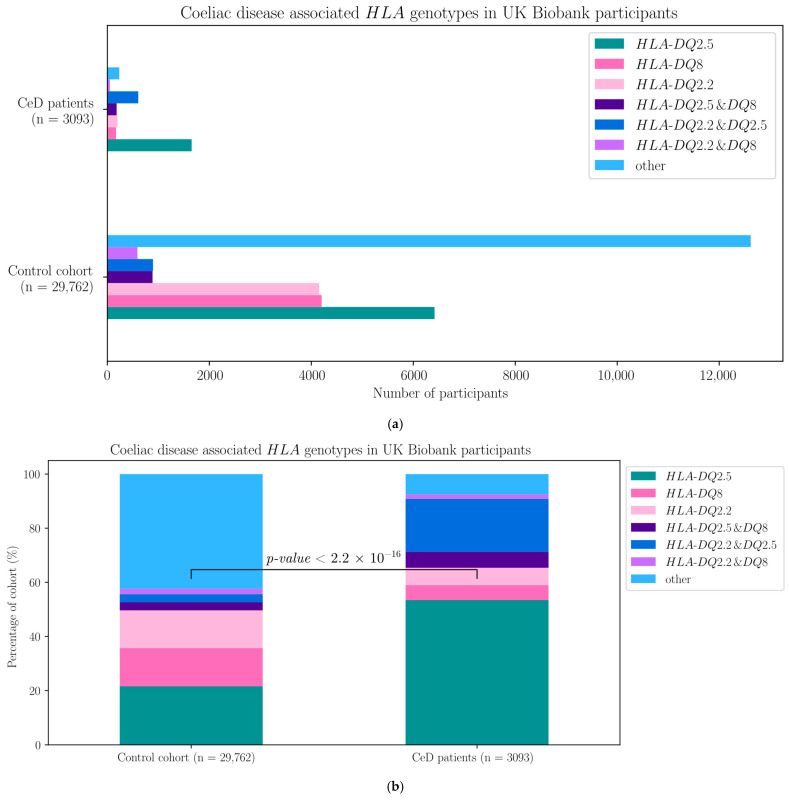
CeD patients (n = 3094) had significantly higher proportions of CeD risk-associated *HLA* genotypes compared to controls (n = 29,762) in the UK Biobank’s 500,000 genome-wide genotyping dataset. The (**a**) number and (**b**) percentage of participants with CeD risk HLA genotypes were significantly more frequent in CeD patients compared to control participants (X-squared = 4062.5, df = 6, *p* < 2.2 × 10^−16^). All participants’ *HLA* genotypes were called using the HLA imputation values provided by the UK Biobank.

**Figure A13:**
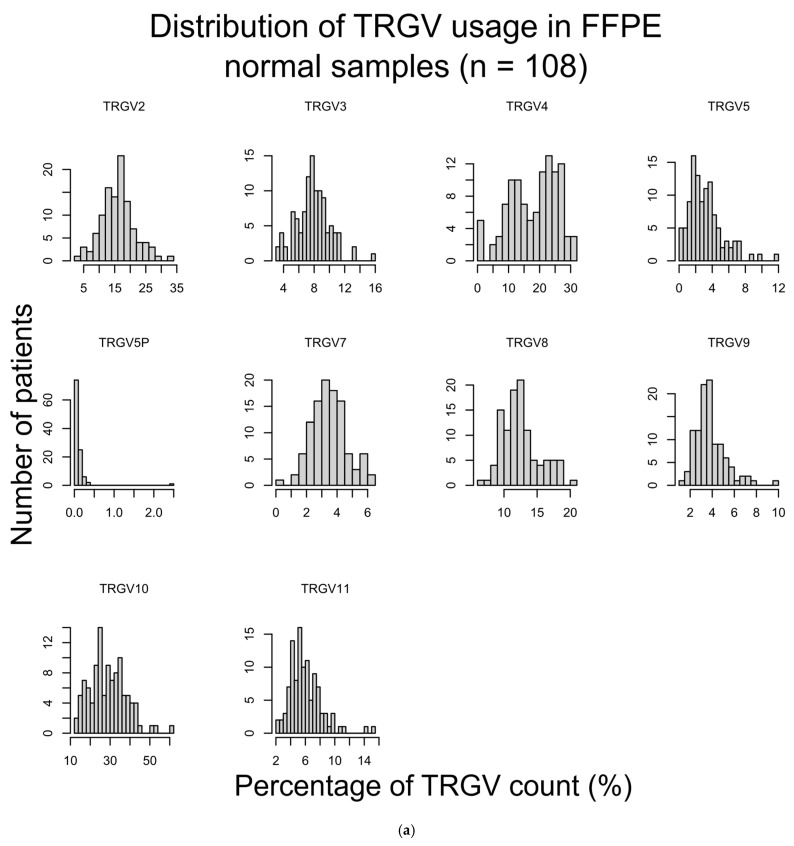

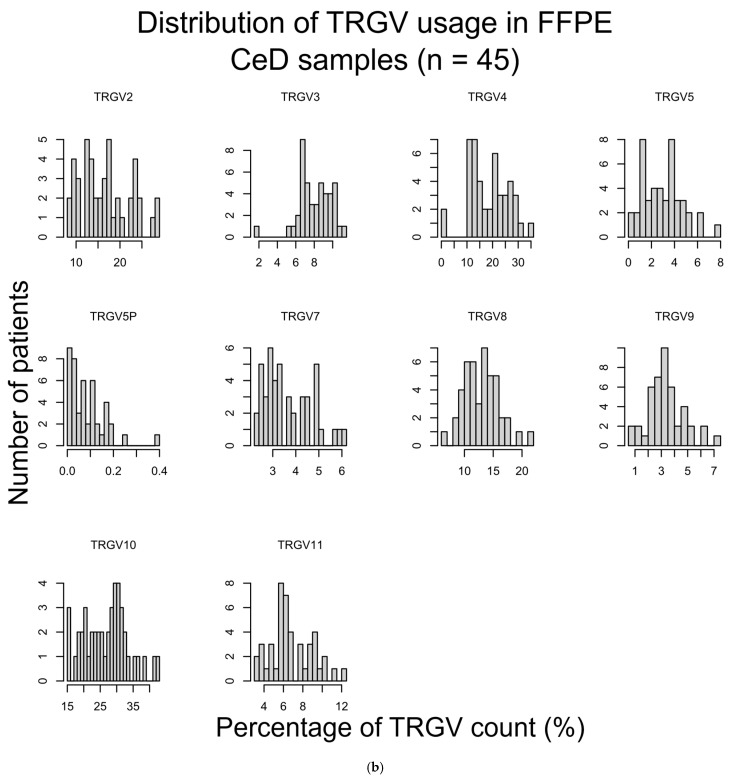
The TRGV usage of (**a**) healthy control (n = 108) and (**b**) CeD FFPE duodenal samples (n = 45) was not normally distributed in the duodenal samples subjected to TRGV usage analysis.

**Figure A14:**
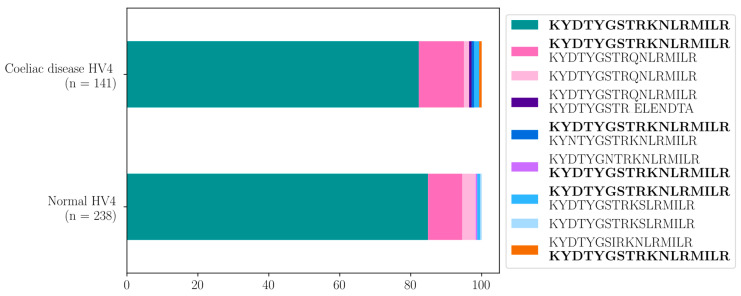
More than 82% of both healthy control and CeD samples were homozygous for the reference HV4 amino acid sequence in the combined cohort. The HV4 analysis was carried out on a cohort of 238 healthy controls and 141 CeD samples. The homozygous reference HV4 sequence was the most common phenotype in both CeD and healthy control samples. Only 10 healthy control and 3 CeD samples did not have any WT HV4 sequences.

The methods of DNA extraction, bulk amplification, and sequencing of the TCR repertoires in Dataset 1 and Dataset 2 were described in Foers et al. [57]. For Dataset 3, the DNA from FFPE duodenal samples and from fresh frozen duodenal samples were extracted using the QiaAmp FFPE DNA kit (Qiagen, Hilden, Germany) and the DNeasy Blood & Tissue Kit (Qiagen), respectively, according to the manufacturer’s instructions (Figure A5).

Hybridisation capture probes were designed for the targeted sequencing of the TCR repertoires of Dataset 3, in collaboration with Nonacus Ltd., Birmingham, UK. Capture probes were designed against the 3′ end of all productive V segments and the 5′ end of all productive J segments available on IMGT, according to their genomic position in the GRCh38.p13 reference genome [82]. Four capture probes (120 bp long) were designed for each productive segment, with the first probe to anneal 10 bp away from the junctional end, with subsequent probes 6 bp away from the previous one.

Samples were prepared for hybridisation capture using the Cell3 Target Library Preparation Kit (b) (Nonacus Ltd., Birmingham, UK), according to the manufacturer’s instructions, and sequenced on an Illumina MiSeq platform.

#### 4.4.2. TRGV and HV4 Analysis Pipeline

The paired-end FASTQ files containing the TRG sequencing data were analysed using MiXCR v4.0.0 (Appendix F) [83,84].

To examine if the TRGV usage was significantly different between CeD and healthy control duodenal samples, pairwise Mann–Whitney U tests were carried out for each of the 10 TRGV segments. To eliminate any false positives due to multiple testing, Bonferroni correction was applied to the *p*-values. For each test, the proportion of the specific TRGV segment was compared between the CeD and the control groups.

The germline HV4 analysis was carried out using Python 3, by identifying variations in the amino acid sequence that directly binds BTNL3 [29,31]. The HV4 was defined as amino acids 10–25 of the FR3, as described by Willcox et al. [31]. The HV4 reference amino acid sequence ‘KYDTYGSTRKNLRMILR’ (named WT sequence for the purposes of this analysis) was demonstrated to be capable of binding BTNL3 [29,31]. Patients were designated as homozygous or heterozygous for the WT amino acid sequence of the HV4 loop, with a minimum of 10% of each HV4 sequence being used as a cutoff percentage for heterozygosity. Fisher’s exact test was applied to compare HV4 WT frequency between CeD and healthy control samples [85].

## Figures and Tables

**Table 1:** Butyrophilins maintain and activate the γδ T cell compartments of mice and humans. Human butyrophilin family members are shortened with all letters capitalised, while only the first letter of mouse butyrophilins is capitalised [24].

	Butyrophilins	γδ T Cell Subset	Role of Butyrophilins	References
**Peripheral blood**	Mouse unidentified	Unidentified	Unidentified	NA
	Alpaca BTN3	Vγ9Vδ2+ T cells	No interaction has been identified	[35,36]
	Human BTN3A homodimers/ heterodimers and BTN2A1 homodimer	Vγ9Vδ2+ T cells	Phosphoantigen-mediated, CDR3-independent γδ T cell activation	[30,33,37,38]
**Skin**	Mouse Skint1 and Skint2	Vγ5Vδ1+ DETC	Thymic selection, tissue homing of dendritic epidermal T cells to the skin	[27,28,32]
	Human?	Vδ1+ T cells	Unidentified, unknown if there is butyrophilin involvement	[39]
**Intestinal epithelium**	Mouse Btnl1 and Btnl6	Vγ7+ IEL	Phenotypic maintenance of the intestinal IEL compartment	[27,28]
	Human BTNL3 and BTNL8	Vγ4Vδ1+ IEL	Phenotypic maintenance of the intestinal IEL compartment	[21,27,29]

**Table 2:** Gene-based burden testing of butyrophilin family non-synonymous coding variants in CeD patients (n = 48) against controls (n = 46) showed significant differences in the disease burden of *BTN2A1* variants. Non-synonymous coding variants that were predicted to be pathogenic or had low minor allele frequencies were considered qualifying variants for burden testing. (a) Burden tests were carried out using the TRAPD program [52] on butyrophilin family qualifying variants in CeD patients (n = 48) against controls (n = 46). Multi-allelic sites were separated into bi-allelic SNPs, as required by the TRAPD documentation [53]. The dominant model defines carriers for gene burden as individuals with at least one qualifying variant within a gene, while the recessive model requires at least two or more qualifying variants. Significant results were highlighted in bold. A version of table (a) with the percentage of individuals and alleles within the CeD and the control groups can be found in Table A10. (b) The *BTN2A1* qualifying SNPs demonstrated a significant burden in CeD samples. Count data of individuals and alleles are in parentheses after the percentage value in columns 6–9 and columns 10–11, respectively. The percentage and count data were calculated from the per sample genotypes found in Table A11.

(a)											
**Gene**	**Qual.** **SNPs**	**CeD N(≥1 HET)**	**CeD N(≥2 HET)**	**CeD N(HOM ALT)**	**CeD Total Allele Count**	**Control N(≥1 HET)**	**Control N(≥2 HET)**	**Control N(HOM ALT)**	**Control Total Allele Count**	**Dominant Model *p*-Value**	**Recessive Model *p*-Value**
***BTN2A1***	3	22	21	3	81	5	4	0	13	**1.46 × 10^−5^**	**3.70 × 10^−8^**
***BTN3A2***	1	5	0	1	7	9	0	1	11	0.929	0.946
***ERMAP***	1	21	0	8	37	20	0	7	34	0.516	0.988
(b) *BTN2A1* variants significantly associated with CeD risk											
**Position (GRCh38)**	**rsID**		**Variation**		**Impact**	**HET CeD**	**HOM ALT CeD**	**HET Control**	**HOM ALT Control**	**Alt Allele in CeD**	**Alt Allele in Controls**
6:26463432	rs13195509		G > A		Missense variant, Val > Met	43.8% (21)	6.3% (3)	8.7% (4)	0.0% (0)	27.1% (26)	4.3% (4)
6:26468098	rs3734542		G > A		Missense variant, Arg > Gln	45.8% (22)	6.3% (3)	10.9% (5)	0.0% (0)	29.2% (28)	5.4% (5)
6:26468317	rs3734543		G > C		Missense variant, Gly > Ala	43.8% (21)	6.3% (3)	8.7% (4)	0.0% (0)	28.1% (27)	4.3% (4)

Abbreviations: Alt allele: alternative or minor allele; CeD: coeliac disease; GRCh38: Genome Reference Consortium Human Build 38; HET: heterozygous; HOM ALT: homozygous for alternative allele; N(≥1 HET): number of individuals carrying at least one heterozygous qualifying variant within the gene; N(≥2 HET): number of individuals carrying at least two heterozygous qualifying variant within the gene; N(HOM ALT): number of individuals carrying at least one homozygous qualifying variant within the gene; qual: qualifying; SNP: single-nucleotide polymorphism.

**Table 3:** SNPs of selected butyrophilin genes present in the UK Biobank.

Gene	SNPs in NCBI	Unique SNPs in NCBI	SNPs in UK Biobank
***BTN2A1***	7912	7605	30
***BTN3A1***	5348	5164	27
***BTN3A2***	5905	5611	21
***BTNL3***	6164	5929	10
***BTNL8***	18,889	18,197	13

**Table 4:** SNPs from *BTN2A1*, *BTN3A1*, and *BTN3A2* genes were significantly associated with CeD status in the UK Biobank. The name of the SNPs in the UK Biobank database is a combination of the reference SNP ID (rsID) from the SNP database (dbSNP) and the reference allele. All *BTN2A1*, *BTN3A1*, *BTN3A2*, *BTNL3*, and *BTNL8* SNPs in the UK Biobank were subjected to single-variant testing to examine their association with CeD. Due to multiple testing, Bonferroni correction was applied. SNPs with a negative ln(OR) are associated with lower CeD risk in this binomial model, meaning that the reference allele is less frequent in CeD patients. SNPs in bold remained significantly associated with CeD in the binomial regression models that also took the *HLA* genotype into account. SNP count and allele count data for the significant SNPs can be found in Table A16. All significant SNPs in control participants were in Hardy–Weinberg equilibrium (Table A17).

Position (GRCh38)	SNP, Reference Allele	Gene	SNP Consequence	CeD Allele Freq	Control Allele Freq	Total Allele Freq	ln(OR)	CeD Risk	Adjusted *p*-Value
**6:26463347**	**rs13195402**	***BTN2A1***	**STOP gained**	**0.768**	**0.892**	**0.880**	**−0.924**	**decrease**	**4.67 × 10^−158^**
**6:26463432**	**rs13195509**	***BTN2A1***	**missense**	**0.754**	**0.879**	**0.867**	**−0.857**	**decrease**	**1.61 × 10^−151^**
6:26475927	rs1407045	*BTN2A1*	intronic	0.584	0.516	0.522	0.273	increase	6.07 × 10^−22^
6:26465807	rs2273558	*BTN2A1*	intronic	0.583	0.677	0.667	−0.396	decrease	1.69 × 10^−41^
6:26460493	rs2893856	*BTN2A1*	intronic	0.113	0.131	0.130	−0.175	decrease	3.23 × 10^−3^
**6:26468098**	**rs3734542**	***BTN2A1***	**missense**	**0.753**	**0.878**	**0.867**	**−0.855**	**decrease**	**8.59 × 10^−151^**
**6:26468317**	**rs3734543**	***BTN2A1***	**missense**	**0.760**	**0.879**	**0.868**	**−0.844**	**decrease**	**1.59 × 10^−140^**
6:26466954	rs3799380	*BTN2A1*	intronic	0.683	0.790	0.780	−0.549	decrease	8.59 × 10^−77^
**6:26474343**	**rs56296968**	***BTN2A1***	**intronic**	**0.696**	**0.807**	**0.796**	**−0.604**	**decrease**	**9.70 × 10^−89^**
6:26456215	rs6456724	*BTN2A1*	2 kb upstream	0.113	0.131	0.130	−0.176	decrease	2.87 × 10^−3^
6:26458037	rs6929846	*BTN2A1*	5′ UTR	0.146	0.174	0.172	−0.206	decrease	3.60 × 10^−6^
6:26473816	rs7773938	*BTN2A1*	intronic	0.696	0.806	0.796	−0.600	decrease	1.15 × 10^−87^
**6:26469647**	**rs9358944**	***BTN2A1***	**intronic**	**0.695**	**0.806**	**0.796**	**−0.604**	**decrease**	**1.55 × 10^−89^**
**6:26471886**	**rs9358945**	***BTN2A1***	**intronic**	**0.694**	**0.806**	**0.796**	**−0.606**	**decrease**	**4.37 × 10^−90^**
6:26404730	rs10456045	*BTN3A1*	intronic	0.596	0.698	0.688	−0.448	decrease	2.97 × 10^−57^
6:26410572	rs1796520	*BTN3A1*	intronic	0.405	0.474	0.467	−0.276	decrease	2.40 × 10^−22^
**6:26404146**	**rs3799378**	***BTN3A1***	**intronic**	**0.653**	**0.762**	**0.752**	**−0.535**	**decrease**	**2.92 × 10^−75^**
6:26405825	rs3857549	*BTN3A1*	intronic	0.948	0.935	0.936	0.221	increase	1.53 × 10^−2^
**6:26409662**	**rs41266839**	***BTN3A1***	**missense**	**0.764**	**0.892**	**0.880**	**−0.924**	**decrease**	**2.12 × 10^−168^**
6:26407180	rs4609015	*BTN3A1*	intronic	0.871	0.854	0.855	0.141	increase	3.82 × 10^−2^
6:26412860	rs6900725	*BTN3A1*	intronic	0.870	0.853	0.855	0.139	increase	4.33 × 10^−2^
6:26401210	rs6912853	*BTN3A1*	2 kb upstream	0.863	0.844	0.846	0.153	increase	7.85 × 10^−3^
6:26413007	rs6920986	*BTN3A1*	intronic	0.870	0.854	0.856	0.138	increase	4.99 × 10^−2^
6:26415409	rs742090	*BTN3A1*	500 b downstream	0.406	0.474	0.468	−0.276	decrease	3.58 × 10^−22^
6:26374321	rs11758089	*BTN3A2*	intronic	0.866	0.844	0.846	0.176	increase	6.30 × 10^−4^
**6:26372558**	**rs12176317**	***BTN3A2***	**intronic**	**0.744**	**0.867**	**0.856**	**−0.809**	**decrease**	**6.72 × 10^−140^**
**6:26366990**	**rs12199613**	***BTN3A2***	**intronic**	**0.514**	**0.612**	**0.602**	**−0.400**	**decrease**	**1.76 × 10^−47^**
**6:26377318**	**rs1977**	***BTN3A2***	**3′ UTR**	**0.740**	**0.864**	**0.853**	**−0.808**	**decrease**	**1.17 × 10^−136^**
**6:26377363**	**rs1979**	***BTN3A2***	**3′ UTR**	**0.743**	**0.867**	**0.855**	**−0.809**	**decrease**	**8.23 × 10^−140^**
**6:26375933**	**rs1985732**	***BTN3A2***	**intronic**	**0.595**	**0.698**	**0.688**	**−0.457**	**decrease**	**2.87 × 10^−59^**
6:26374430	rs2073526	*BTN3A2*	intronic	0.370	0.442	0.435	−0.295	decrease	9.15 × 10^−25^
**6:26363527**	**rs9358934**	***BTN3A2***	**2 kb upstream**	**0.744**	**0.866**	**0.855**	**−0.803**	**decrease**	**2.34 × 10^−137^**
**6:26364702**	**rs9379855**	***BTN3A2***	**2 kb upstream**	**0.743**	**0.866**	**0.855**	**−0.804**	**decrease**	**8.85 × 10^−138^**
**6:26367461**	**rs9379858**	***BTN3A2***	**intronic**	**0.743**	**0.866**	**0.855**	**−0.802**	**decrease**	**3.19 × 10^−137^**
**6:26369321**	**rs9379859**	***BTN3A2***	**intronic**	**0.744**	**0.867**	**0.855**	**−0.803**	**decrease**	**5.37 × 10^−137^**
**6:26373450**	**rs9393713**	***BTN3A2***	**intronic**	**0.743**	**0.868**	**0.856**	**−0.814**	**decrease**	**1.07 × 10^−141^**
**6:26373512**	**rs9393714**	***BTN3A2***	**intronic**	**0.743**	**0.868**	**0.856**	**−0.813**	**decrease**	**6.99 × 10^−141^**

Abbreviations: CeD: coeliac disease; freq: frequency; GRCh38: Genome Reference Consortium Human Build 38; kb: kilobase; ln(OR): natural logarithm of the odds ratio; SNP: single-nucleotide polymorphism; UTR: untranslated region.

**Table 5:** Twenty SNPs from *BTN2A1*, *BTN3A1*, and *BTN3A2* genes were significantly associated with CeD status in the UK Biobank when *HLA* genotypes were included in the single-variant testing models. *BTN2A1*, *BTN3A2*, *BTNL3*, and *BTNL8* SNPs in the UK Biobank were subjected to single-variant testing to examine their association with CeD. Due to multiple testing, Bonferroni correction was applied. SNPs with a negative ln(OR) are associated with lower CeD risk in this binomial model. All significant SNPs in control participants were in Hardy–Weinberg equilibrium (Table A19).

SNP, Reference Allele	Gene	SNP Consequence	ln(OR)	CeD Risk	Adjusted *p*-Value
rs13195402	*BTN2A1*	STOP gained	−0.20727	decrease	8.15 × 10^−6^
rs13195509	*BTN2A1*	missense	−0.19239	decrease	1.62 × 10^−5^
rs3734542	*BTN2A1*	missense	−0.18831	decrease	2.94 × 10^−5^
rs3734543	*BTN2A1*	missense	−0.16744	decrease	8.23 × 10^−4^
rs56296968	*BTN2A1*	intronic	−0.11753	decrease	4.20 × 10^−2^
rs9358944	*BTN2A1*	intronic	−0.11786	decrease	3.83 × 10^−2^
rs9358945	*BTN2A1*	intronic	−0.12018	decrease	2.91 × 10^−2^
rs3799378	*BTN3A1*	intronic	−0.14327	decrease	7.04 × 10^−4^
rs41266839	*BTN3A1*	missense	−0.21469	decrease	1.06 × 10^−6^
rs12176317	*BTN3A2*	intronic	−0.1974	decrease	3.50 × 10^−6^
rs12199613	*BTN3A2*	intronic	−0.12296	decrease	3.31 × 10^−3^
rs1977	*BTN3A2*	3′ UTR	−0.20238	decrease	2.06 × 10^−6^
rs1979	*BTN3A2*	3′ UTR	−0.19756	decrease	3.40 × 10^−6^
rs1985732	*BTN3A2*	intronic	−0.10975	decrease	3.35 × 10^−2^
rs9358934	*BTN3A2*	2 kb upstream	−0.19286	decrease	7.53 × 10^−6^
rs9379855	*BTN3A2*	2 kb upstream	−0.19406	decrease	6.04 × 10^−6^
rs9379858	*BTN3A2*	intronic	−0.19156	decrease	8.99 × 10^−6^
rs9379859	*BTN3A2*	intronic	−0.19261	decrease	8.10 × 10^−6^
rs9393713	*BTN3A2*	intronic	−0.2056	decrease	9.27 × 10^−7^
rs9393714	*BTN3A2*	intronic	−0.20087	decrease	2.08 × 10^−6^

Abbreviations: CeD: coeliac disease; ln(OR): natural logarithm of the odds ratio; SNP: single-nucleotide polymorphism.

**Table 6:** Single-variant testing in *HLA*-matched groups from the UK Biobank dataset only identified significant SNPs associated with CeD status in individuals with *HLA-DQ2.5* genotypes. The CeD and control participants of the UK Biobank dataset were divided into *HLA*-matched case-control groups for single-variant testing. The association between *BTN2A1*, *BTN3A1*, *BTN3A2*, *BTNL3*, and *BTNL8* SNPs and CeD status was investigated. Significant association between the SNPs and CeD status was only present in *HLA-DQ2.5*-matched individuals (in bold).

*HLA* Genotype of Individuals in Model	Number of CeD Participants	Number of Controls	Number of Significant SNPs
*HLA-DQ2.2*	199	4154	0
***HLA-DQ2.5***	**1652**	**6416**	**21**
*HLA-DQ8*	171	4203	0
*HLA-DQ2.2*, *HLA-DQ2.5*	606	895	0
*HLA-DQ2.2*, *HLA-DQ8*	50	590	0
*HLA-DQ2.5*, *HLA-DQ8*	182	886	0
Other	234	12,618	0

**Table 7:** Butyrophilin SNPs only remained significantly associated with CeD status in the *HLA-DQ2.5*-restricted UK Biobank analysis. The name of the SNPs in the UK Biobank database is a combination of the SNP name and the reference allele. All *BTN2A1*, *BTN3A1*, *BTN3A2*, *BTNL3*, and *BTNL8* SNPs in the UK Biobank were subjected to single-variant testing to examine their association with CeD. Due to multiple testing, Bonferroni correction was applied. SNPs with a negative ln(OR) are associated with lower CeD risk in this binomial model. The SNP in bold was a novel SNP significantly associated with CeD unique to the *HLA-DQ2.5* model, while the other SNPs were also significant in the non-*HLA* and the *HLA* models. SNP count and allele count data for the significant SNPs can be found in Table A21. The allele frequency of all the significantly associated SNPs in the control group significantly differed from the Hardy–Weinberg equilibrium (Table A22).

SNP, Reference Allele	Gene	SNP Consequence	CeD Allele Freq	Control Allele Freq	Total Allele Freq	ln(OR)	CeD Risk	Adjusted *p*-Value
rs13195402	*BTN2A1*	STOP gained	0.704	0.751	0.741	−0.27812	decrease	5.10 × 10^−7^
rs13195509	*BTN2A1*	missense	0.687	0.734	0.724	−0.25542	decrease	1.75 × 10^−6^
rs3734542	*BTN2A1*	missense	0.687	0.733	0.723	−0.25235	decrease	2.52 × 10^−6^
rs3734543	*BTN2A1*	missense	0.695	0.736	0.728	−0.23279	decrease	5.43 × 10^−5^
rs56296968	*BTN2A1*	intronic	0.640	0.673	0.666	−0.15928	decrease	2.33 × 10^−2^
**rs7773938**	***BTN2A1***	**intronic**	**0.640**	**0.672**	**0.666**	**−0.15755**	**decrease**	**2.66 × 10^−2^**
rs9358944	*BTN2A1*	intronic	0.638	0.671	0.664	−0.16238	decrease	1.61 × 10^−2^
rs9358945	*BTN2A1*	intronic	0.638	0.671	0.665	−0.16499	decrease	1.26 × 10^−2^
rs3799378	*BTN3A1*	intronic	0.596	0.637	0.628	−0.18712	decrease	8.10 × 10^−4^
rs41266839	*BTN3A1*	missense	0.697	0.747	0.737	−0.28267	decrease	7.25 × 10^−8^
rs12176317	*BTN3A2*	intronic	0.679	0.729	0.719	−0.26575	decrease	2.82 × 10^−7^
rs12199613	*BTN3A2*	intronic	0.459	0.500	0.491	−0.1717	decrease	2.06 × 10^−3^
rs1977	*BTN3A2*	3′ UTR	0.676	0.726	0.716	−0.268	decrease	2.99 × 10^−7^
rs1979	*BTN3A2*	3′ UTR	0.679	0.728	0.718	−0.26376	decrease	3.63 × 10^−7^
rs1985732	*BTN3A2*	intronic	0.539	0.574	0.567	−0.15063	decrease	2.35 × 10^−2^
rs9358934	*BTN3A2*	2 kb upstream	0.680	0.729	0.719	−0.25825	decrease	8.85 × 10^−7^
rs9379855	*BTN3A2*	2 kb upstream	0.680	0.728	0.718	−0.25967	decrease	6.76 × 10^−7^
rs9379858	*BTN3A2*	intronic	0.680	0.728	0.718	−0.25566	decrease	1.18 × 10^−6^
rs9379859	*BTN3A2*	intronic	0.681	0.729	0.719	−0.26105	decrease	6.31 × 10^−7^
rs9393713	*BTN3A2*	intronic	0.678	0.729	0.719	−0.27313	decrease	1.06 × 10^−7^
rs9393714	*BTN3A2*	intronic	0.679	0.729	0.719	−0.26778	decrease	2.25 × 10^−7^

Abbreviations: CeD: coeliac disease; freq: frequency; ln(OR): natural logarithm of the odds ratio; SNP: single-nucleotide polymorphism.

**Table 8:** The coeliac disease and healthy control patient TRG datasets analysed for TRGV usage and HV4 sequence variations. FFPE: formalin-fixed, paraffin-embedded.

	Coeliac Disease	Healthy Control	Sequencing Method
Dataset 1	34 FFPE, 12 fresh frozen duodenal	97 FFPE duodenal	Lymphotrack (Invivoscribe Inc., San Diego, CA, USA) and Illumina Miseq micro (San Diego, CA, USA)
Dataset 2	11 FFPE duodenal	11 FFPE duodenal	Lymphotrack (Invivoscribe Inc.) and Illumina Miseq
Dataset 3	84 blood	130 blood	Illumina NextSeq
Combined	84 blood, 48 FFPE duodenal, 12 fresh frozen duodenal	130 blood, 108 FFPE duodenal	NA

**Table 9:** More than 95% of participants possessed at least one reference HV4 loop regardless of their CeD status. The dataset consisted of 238 healthy controls and 141 CeD samples. (a) Seven unique HV4 amino acid sequences were identified in the dataset. (b) The homozygous WT HV4 phenotype was the most frequent in both the healthy control and CeD groups.

(a)					
**HV4 Amino Acid Sequence**	**Amino Acid Change**	**Effect**	**Freq. in Healthy Control Samples (n = 238)**	**Freq. in CeD Samples (n = 141)**	**Predicted Change in Binding** [31]
**KYDTYGSTRKNLRMILR (WT)**	-	-	430/476 = 0.903	254/282 = 0.901	-
**KYDTYGSTRQNLRMILR**	Lysine > Glutamine	Positive charge > polar uncharged	41/476 = 0.086	23/282 = 0.082	Marginal reduction in binding
**KYDTYGSTRKSLRMILR**	Asparagine > Serine	Polar uncharged > polar uncharged	4/476 = 0.008	2/282 = 0.007	Unknown
**KYDTYGSTR_ELENDTA**	Lysine > frameshift	Positive charge > different sequence	0	1/282 = 0.003	Unknown
**KYNTYGSTRKNLRMILR**	Aspartic acid > Asparagine	Negative charge > polar uncharged	0	1/282 = 0.003	Disrupted binding
**KYDTYGNTRKNLRMILR**	Serine > Asparagine	Polar uncharged > polar uncharged	1/476 = 0.002	0	Unknown
**KYDTYGSIRKNLRMILR**	Threonine > Isoleucine	Polar uncharged > apolar	0	1/282 = 0.003	Unknown
(b)					
**Phenotype**		**Combined Healthy Control Samples (n = 238)**		**Combined CeD Samples (n = 141)**	
**WT**		202		116	
**WT, KYDTYGSTRQNLRMILR**		23		18	
**KYDTYGSTRQNLRMILR**		9		2	
**WT, KYDTYGSTRKSLRMILR**		2		2	
**KYDTYGSTRQNLRMILR, KYDTYGSTR_ELENDTA**		0		1	
**WT, KYNTYGSTRKNLRMILR**		0		1	
WT, KYDTYGNTRKNLRMILR		1		0	
KYDTYGSTRKSLRMILR		1		0	
WT, KYDTYGSIRKNLRMILR		0		1	

**Figure A2:**
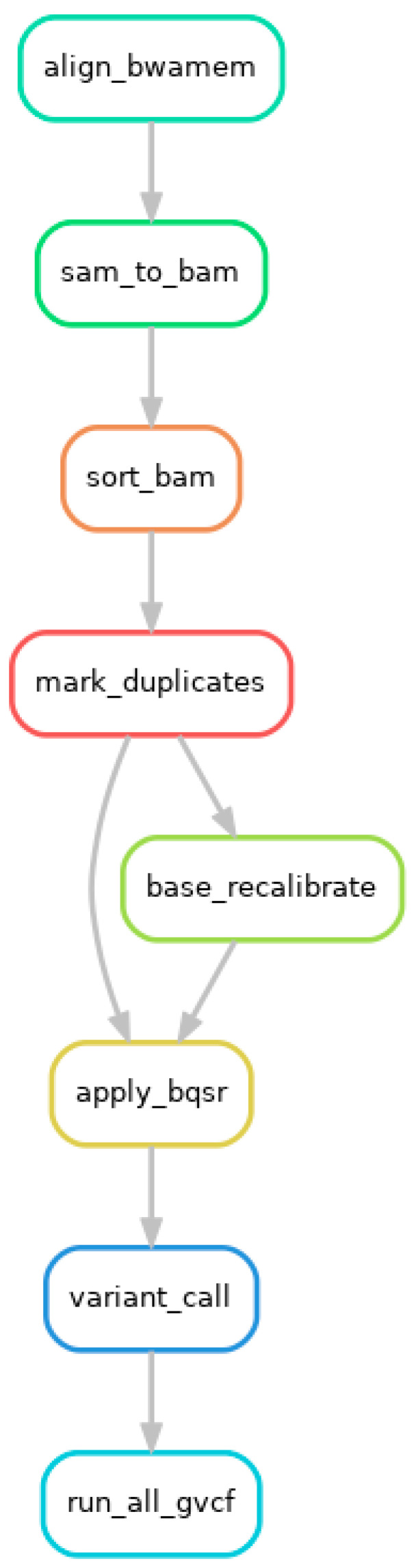
The genetic variants were called per sample for the hybridisation capture samples by adapting GATK best practices. Each box symbolises a step or rule in the Snakemake workflow [87]. The directed acyclic graph was created using the Snakemake software’s built-in commands.

**Figure A3:**
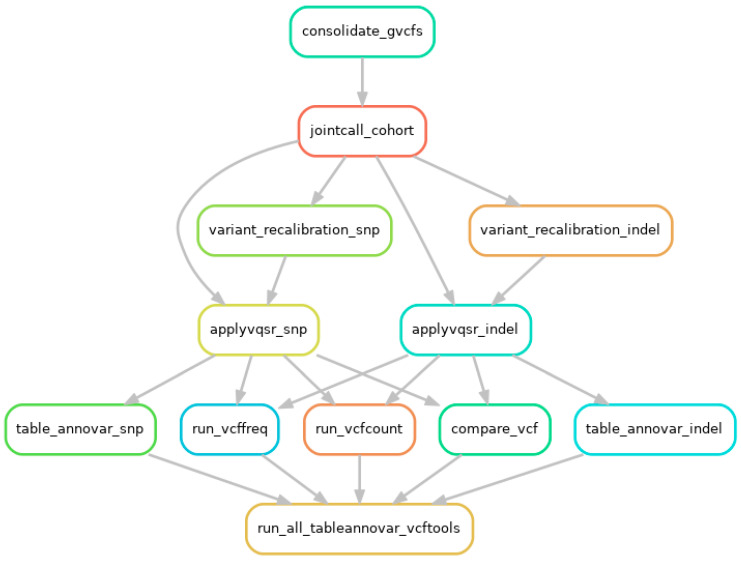
The samples of the targeted sequencing cohort (n = 94) were jointly genotyped, and the variants were annotated using an adapted GATK workflow. Each box symbolises a step or rule in the Snakemake workflow [87]. The directed acyclic graph was created using the Snakemake software’s built-in commands.

**Figure A8:**
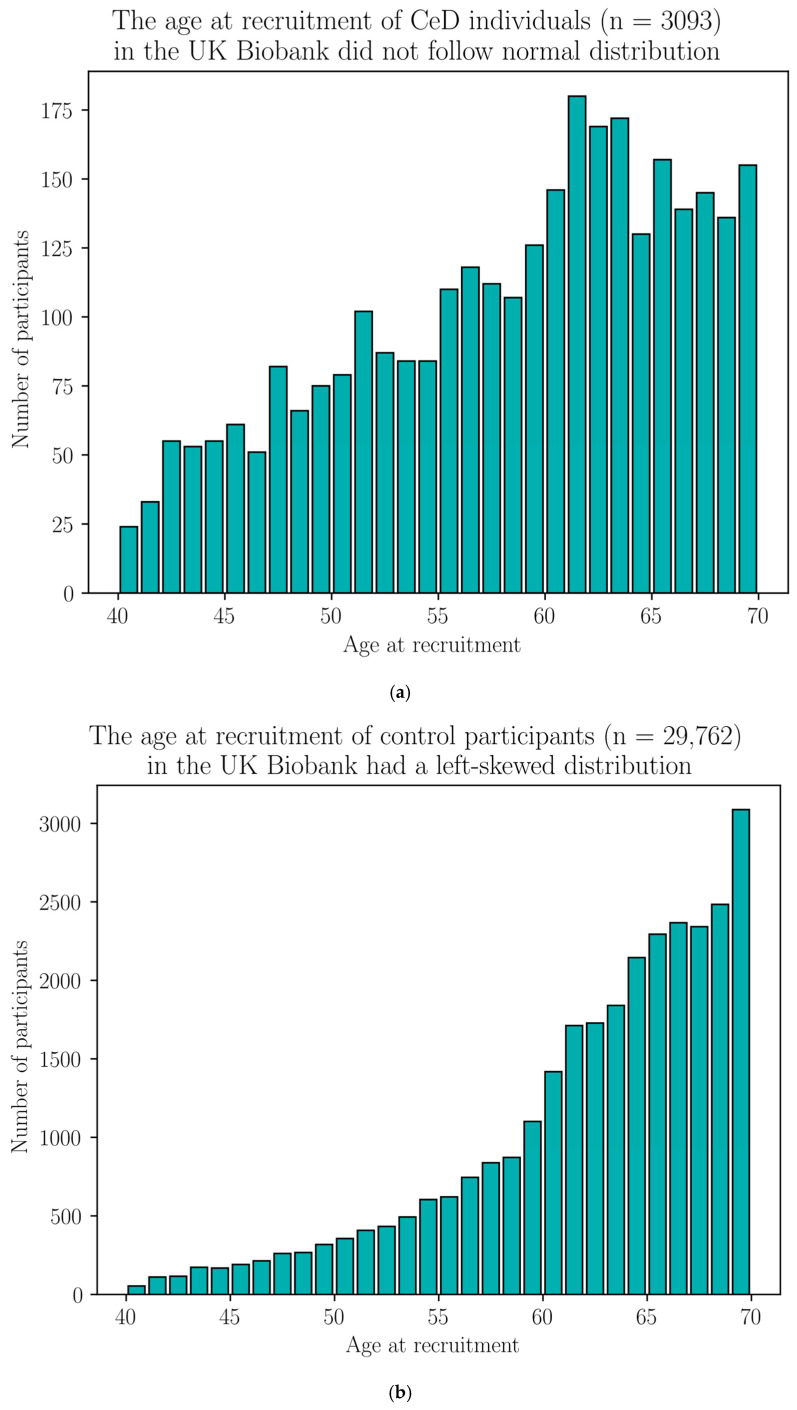

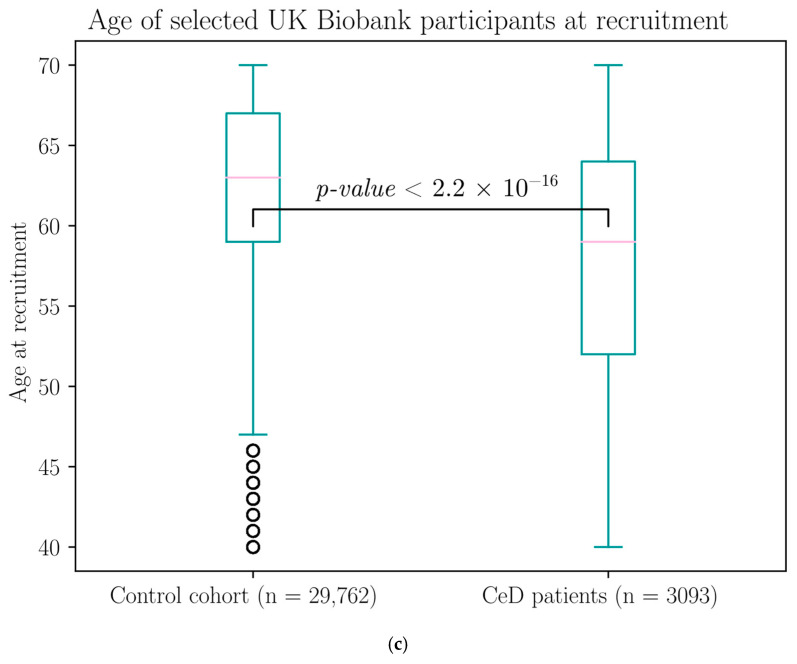
CeD patients were significantly younger than control individuals when they were recruited for the initial UK Biobank study. The distribution of the age at recruitment of (**a**) 29,762 control participants and (**b**) 3094 CeD patients from the UK Biobank dataset did not follow a normal distribution. (**c**) The mean age at recruitment was significantly higher in control individuals (t = 27.297, df = 3539.3, *p*-value < 2.2 × 10^−16^).

**Figure A9:**
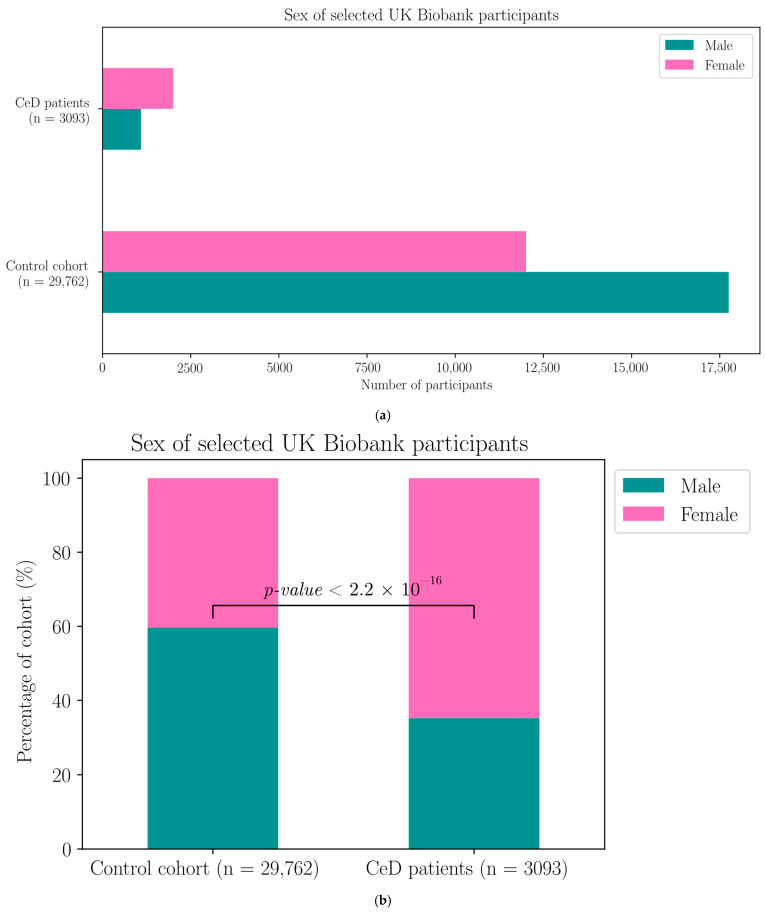
A significantly higher proportion of individuals diagnosed with CeD were female participants, compared with the control cohort in the UK Biobank dataset. The sex of 3094 CeD patients and 29,762 control participants was analysed from the UK Biobank. The figure shows the (**a**) number and (**b**) percentage of each sex for CeD and control participants. The CeD group in the UK Biobank had a significantly higher proportion of female participants (X-squared = 683.91, df = 1, *p* < 2.2 × 10^−16^).

**Figure A10:**
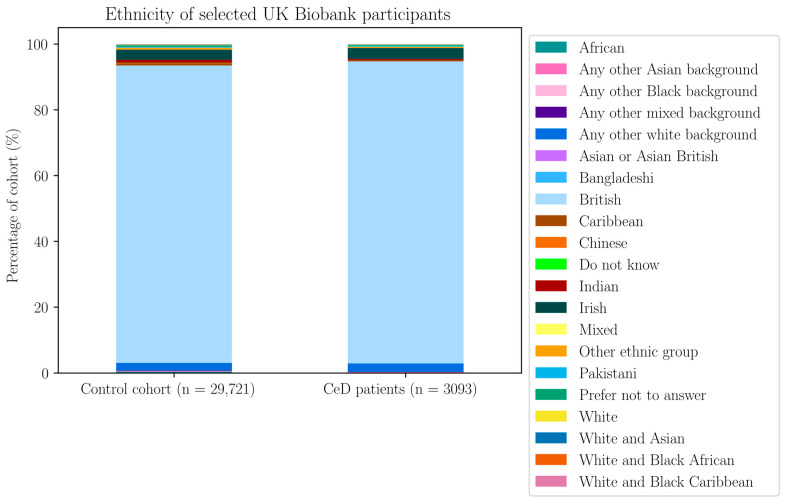
White British was the most common ethnic background in the UK Biobank participants, regardless of CeD status. The ethnic backgrounds of 3094 CeD patients and 29,762 control participants were analysed from the UK Biobank. There were no significant differences in the ethnic background of CeD and control participants after Bonferroni correction.

**Figure A11:**
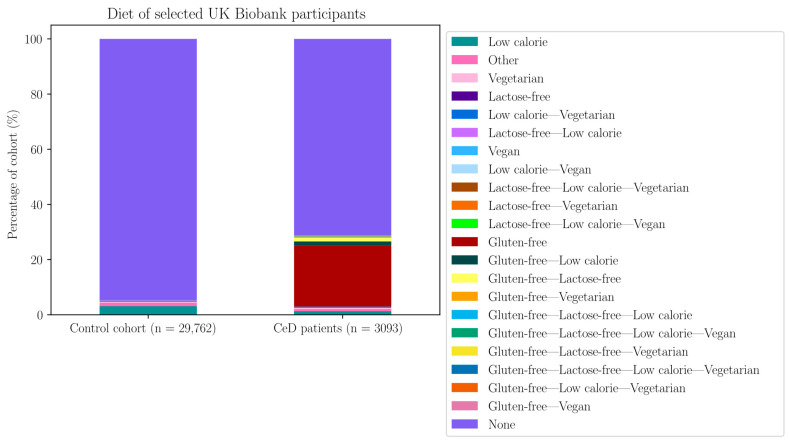
The majority of UK Biobank participants reported no special diet in both control and CeD groups. The diet of 3094 CeD patients and 29,762 control participants was analysed from the UK Biobank. The majority of UK Biobank participants reported no special diet in both control (94.7%, 28,185/29,762) and CeD (71.2%, 2204/3094) groups. When participants following a gluten-free diet were excluded from the analysis, the proportions of participants following each special diet were significantly different between the CeD and control groups (X-squared = 23.303, df = 11, *p* = 0.01602). This was likely caused by having to exclude patients who followed multiple diets that included a gluten-free diet. Participants on a gluten-free diet were excluded from the control group.

## Data Availability

Targeted sequencing data of selected butyrophilin family genes in a cohort of 94 samples (2.1): https://zenodo.org/records/15203243 (accessed on 12 April 2025). γδ TCR sequencing data of 46 CeD and 97 healthy control duodenal samples (Dataset 1, Table 2): https://dataview.ncbi.nlm.nih.gov/object/PRJNA1330789?reviewer=sp7hjkohgpbo6mt3qv7thd57fp (accessed on 26 October 2025). γδ TCR sequencing data of 11 CeD and 11 healthy control duodenal samples (Dataset 2, Table 2): https://dataview.ncbi.nlm.nih.gov/object/PRJNA1330746?reviewer=dcl0ipo3ftc3m83b637i0hjmpd (accessed on 26 October 2025). γδ TCR sequencing data of 84 CeD and 130 healthy control blood samples (Dataset 3, Table 2): https://dataview.ncbi.nlm.nih.gov/object/PRJNA1330754?reviewer=uoqvavqemvh35apdc0ifn99585 (accessed on 26 October 2025).

## Abbreviations

The following abbreviations are used in this manuscript:

BTN/BTNL	Butyrophilin/butyrophilin-like
CeD	Coeliac disease
CNV	Copy number variation
DC	Dendritic cell
GFD	Gluten-free diet
FFPE	Formalin-fixed, paraffin-embedded
HLA	Human leukocyte antigen
HPA	Human Protein Atlas
HV4	Hypervariable region 4
IEL	Intraepithelial lymphocyte
NK cell	Natural Killer cell
TRGV	T cell receptor γ variable region
SNP/SNV	Single-nucleotide polymorphism/variation
WT	Wild-type

## Appendix A. The Molecular Background of the 56 kb *BTNL3*BTNL8* Deletion Variant

The *BTNL3* and *BTNL8* loci are segmental duplications and share a high sequence similarity. During meiosis, highly identical sequences are prone to recombination, which can give rise to CNVs. This is the likely explanation for the *BTNL8*BTNL3* 56 kb deletion copy number described by Aigner et al. [41]. This study reported that 58.4% of their 346 samples of European ancestry had at least one *BTNL8*BTNL3* deletion allele (Table A1). This CNV has been shown to encode a BTNL8*3 fusion protein, which consists of the transmembrane domain, the extracellular IgV and IgC domains of BTNL8, and the intracellular signalling domain of BTNL3. As the BTNL3-IgV domain is missing in the fusion protein, it is plausible that the BTNL8*3 fusion protein has an impaired ability to bind to the Vγ4Vδ1+ T cells in the small intestine [31].

**Table A1:** The *BTNL8*BTNL3* copy number variation is present in 58.4% of individuals of European ancestry, as first described by Aigner et al. [41]. Carriers are defined as individuals with at least one *BTNL8*BTNL3* deletion allele. Abbreviations: CEU: Utah residents with Northern and Western European ancestry; HapMap: International HapMap Project; het.: heterozygous; HGDP: Human Genome Diversity Panel; hom.: homozygous; N: number.

	Population	Hom. for Deletion	Het. for Deletion	Hom. for Full Sequences	Deletion Allele N	Deletion Allele Frequency	Group N	Carriers N	Carriers %
**HapMap**	CEU	17	56	68	90	0.319	141	73	51.8
	Toskani, Italia	9	45	34	63	0.358	88	54	61.4
**HGDP**	France	7	28	17	42	0.404	52	35	67.3
	Italy	5	18	13	28	0.389	36	23	63.9
	Italy (Bergamo)	3	2	9	8	0.286	14	5	35.7
	Orkney Islands	1	11	3	13	0.433	15	12	80.0
**Total**	**European ancestry**	**42**	**160**	**144**	**244**	**0.353**	**346**	**202**	**58.4**

## Appendix B. Selecting and Sequencing the Butyrophilin Genes of Interest

### Appendix B.1. HPA Expression Profiles of Butyrophilin Family Genes

**Table A2:** The expression of the butyrophilin family members in intestinal tissues provided by the HPA. Butyrophilin protein expression in (a) the duodenum and in (b) the small intestine, colon, and rectum was extracted from the Tissue section of the Human Protein Atlas database [64,86].

(a) Butyrophilin family expression in the duodenum						
	**Reliability as Defined by the HPA**	**Protein Expression in Duodenum (IHC)** ***If RNA Data Only***	**Comment**			**Included?**
**ERMAP**	Uncertain	High	Uncertain Tissue Atlas reliability score High expression in digestive tissues Low–medium expression in immune cells			Yes
**MOG**	Enhanced	None	Not expressed in immune cells or digestive tissues Genomic variance control for the significance of butyrophilin variation in CeD risk			Yes
**BTN1A1**	Supported	None	Not expressed in digestive tissues or immune cells			No
**BTN2A1**	Approved	Medium	Included due to reliability score and expression Implicated in the stimulation of Vγ9Vδ2+ T cells [37]			Yes
**BTN2A2**	Uncertain	High	Uncertain Tissue Atlas reliability score Medium–high expression in digestive tissues Expressed in immune cells			Yes
**BTN3A1**	Approved	Medium	Included due to reliability score and expression Implicated in the stimulation of Vγ9Vδ2+ T cells [30]			Yes
**BTN3A2**	Uncertain	Medium	Uncertain Tissue Atlas reliability score Implicated in the stimulation of Vγ9Vδ2+ T cells [27]			Yes
**BTN3A3**	Enhanced	Medium	Included due to reliability score and expression			Yes
***BTNL2***	Pending	*None*	Pending Tissue Atlas reliability score *HLA*-independent significant association with CeD [19]			Yes
***BTNL3***	Pending	*High*	Pending Tissue Atlas reliability score No protein expression data available for the intestinal tissues Previously documented role in CeD [21]			Yes
**BTNL8**	Enhanced	Medium	Previously documented role in CeD [21]			Yes
***BTNL9***	Pending	*Low*	Pending Tissue Atlas reliability score No protein expression data available for the intestinal tissues Not expressed in immune cells			No
**BTNL10**	NA	NA	No entry			No
**SKINT1L**	NA	NA	No entry			No
**BTN2A3P**	NA	NA	No entry			No
(b) Expression of selected butyrophilin family members in the small intestine, colon, and rectum						
**Tissue Expression (IHC) *If RNA Data Only***						**Included in the Panel?**
	**Reliability as Defined by the HPA**	**Small Intestine** **(Glandular)**	**Duodenum** **(Glandular)**	**Rectum** **(Glandular)**	**Colo** **(Glandular)**	
ERMAP	Uncertain	High	High	High	High	Yes
MOG	Enhanced	none	none	none	none	Yes
BTN2A1	Approved	Low	Medium	Medium	Medium	Yes
BTN2A2	Uncertain	High	High	Medium	Medium	Yes
BTN3A1	Approved	High	Medium	High	High	Yes
BTN3A2	Uncertain	High	Medium	Medium	Medium	Yes
BTN3A3	Enhanced	Medium	Medium	Medium	Medium	Yes
*BTNL2*	Pending	*Very low*	none	none	none	Yes
*BTNL3*	Pending	*High*	*High*	*High*	*High*	Yes
BTNL8	Enhanced	Medium	Medium	none	none	Yes

The reliability score of each entry was provided by the HPA. The score was based on the reliability between the RNA sequencing and antibody staining data. For most genes, the HPA provided immunohistochemical evidence for the protein expression of the genes. Only RNA sequencing data were available for *BTNL2*, *BTNL3*, and *BTNL9* expression, denoted with *. Evidence linking the butyrophilins to immune cell function and CeD risk was also used to determine inclusion in the custom sequencing panel [19,21,30]. The expression of butyrophilin family members in (b) is shown only for the genes that were selected for the custom probe panel [data accessed in 2021].

**Table A3:** The expression of butyrophilin family genes of interest in immune cells provided by the HPA. The butyrophilin family mRNA expression data in immune cells were accessed from the Human Protein Atlas (HPA) [64,86].

Immune Cell Expression (RNA Sequencing)								Included in the Panel?
	Reliability as Defined by the HPA	γδ T Cells	T Cells	T-Reg	DCs	Macrophages	NK Cells	
*ERMAP*	Uncertain	Low	Medium	Medium	Medium	Medium	Low	Yes
*MOG*	Enhanced	Very low	None	Very low	None	None	None	Yes
*BTN2A1*	Approved	Medium	Medium	Medium	Medium	High	Low	Yes
*BTN2A2*	Uncertain	Low	Medium	Medium	High	High	Medium	Yes
*BTN3A1*	Approved	High	High	High	Low	Medium	High	Yes
*BTN3A2*	Uncertain	High	High	High	Medium	High	High	Yes
*BTN3A3*	Enhanced	High	High	High	Medium	Medium	High	Yes
*BTNL2*	Pending	None	None	None	None	None	None	Yes
*BTNL3*	Pending	None	None	None	None	None	None	Yes
*BTNL8*	Enhanced	None	None	None	None	None	None	Yes

The reliability score of each entry was provided by the HPA. The score was based on the reliability between the RNA sequencing and antibody staining data. The RNA expression data from T cells, DCs, NK cells, and macrophages were selected from the Single cell type section of the gene entries. The RNA expression data from T-regs and γδ T cells were accessed from the Immune cell type section of the gene entries [data accessed in 2021].

**Table A4:** The GRCh38.p12 genomic location of the selected butyrophilin genes.

Gene of Interest	Location (GRCh38.p12)
*BTN2A1*	chr6:26,457,955–26,476,622
*BTN2A2*	chr6:26,382,893–26,394,874
*BTN3A1*	chr6:26,402,269–26,415,216
*BTN3A2*	chr6:26,365,170–26,378,320
*BTN3A3*	chr6:26,440,504–26,453,415
*BTNL2*	chr6:32,393,339–32,408,879
*BTNL3*	chr5:180,988,846–181,006,727
*BTNL8*	chr5:180,899,097–180,952,166
*ERMAP*	chr1:42,817,122–42,844,991
*MOG*	chr6:29,657,092–29,672,365

### Appendix B.2. Modified Nonacus Cell3 Hybridisation Capture and Illumina Sequencing

To summarise the modified protocol, the DNA quality of all samples was measured initially, to acquire 200 ng input DNA for the fragmentation step (1.B) of the hybridisation capture protocol (Figure A1). Fragmentation time in step 1.B was modified to 30 min to achieve 200 bp DNA fragments. Afterwards, the Genomic Tapestation kit (Agilent Technologies, Santa Clara, CA, USA) was used to check the correct fragment size.

Next, in step 1.C, unique molecular identifier (UMI) adapters were ligated to the DNA fragments. During the magnetic bead clean up using NGS Target Pure Clean-Up Beads (Nonacus), the adapter-ligated DNA fragments were incubated for 6 min on the magnetic strip. Nuclease-free water was used in the final step of the clean up.

The pre-hybridisation PCR in step 1.D was carried out for 4 cycles. Afterwards, the DNA concentration of each reaction was measured in step 1.E. Samples with DNA concentrations lower than 10 ng/μL were subjected to additional rounds of amplification, and steps 1.D and 1.E were repeated. CB22, CB24, CB26, CB27, and CB30 had low DNA concentration after the amplification step; therefore, they were subjected to 5 additional cycles of PCR. Sample NB7 was also subjected to 6 more cycles of PCR.

In step 2.A, the samples were pooled together, each library containing DNA fragments from 8 patients. For each sample, 125 ng of DNA was used, and the pooled libraries were dried using a vacuum concentrator for 30 min. Each pooled library was hybridised overnight at 65 °C with the designed butyrophilin probes and a 1:50 dilution of *HLA* probes provided by Nonacus.

In step 2.B, the hybridised library was captured on Dynabeads M-270 Streptavidin beads (Invitrogen/Thermo Fisher Scientific, Waltham, MA, USA). Post-hybridisation PCR was carried out for 20 cycles in step 2.C. This was followed by bead clean up using NGS Target Pure Clean-Up Beads. Similar to step 1.C, the beads and the amplified captured library were incubated for 6 min on the magnetic strip. Nuclease-free water was used in the final step of the clean up.

In step 2D, the concentration of the captured library was measured using Qubit (CAT Q32851, lot 2313066), and the size of the DNA fragments in each hybridisation library was quantified using the 4200 Tapestation (CAT G2991A, lot DEDAA01701).

Illumina MiSeq sequencing required each captured hybridisation library to be diluted to 10 nM concentration. The concentration of each sample in nM was calculated using the following equation:

$$concentration\;\left(nM\right)=\frac{concentration\;(ng/\mu L)}{660\times DNA\;fragment\;size\;(bp)}\times 10^{6}$$

where the concentration (ng/μL) was the DNA concentration of the captured library as quantified by Qubit, and the DNA fragment size (bp) was the average DNA fragment size as measured by Tapestation.

After each of the 12 hybridised libraries was diluted to 10 nm, 2 μL of each diluted library was mixed together. Afterwards, 10 μL was sent to the Department of Biochemistry, University of Cambridge, UK for sequencing using the Illumina MiSeq system (San Diego, CA, USA).

### Appendix B.3. Measuring DNA Quantity and Fragment Size

A Qubit 2.0 fluorometer (Invitrogen) was used to measure nucleic acid quantity, using the Qubit dsDNA High Sensitivity Quantification Assay kit (Invitrogen) according to the manufacturers’ instructions.

The 4200 Tapestation System (Agilent Technologies) was used to measure the fragment size of DNA samples. The Genomic DNA ScreenTape Analysis kit (Agilent Technologies) was used to measure the fragment sizes of DNA samples after step 1.B of the Nonacus hybridisation capture protocol (Nonacus). The D1000 ScreenTape Assay kit (Agilent Technologies) was used to measure the DNA sizes of the pooled hybridisation libraries in step 2.D of the Nonacus hybridisation capture protocol.

## Appendix C. Detailed Germline Short-Variant Discovery Protocol

### Appendix C.1. Per Sample Preprocesses and Variant Call Using GATK

The variant discovery process for the targeted sequencing cohort was split into two parts. In the first part, each patient sample was processed separately. The variants were called per sample as recommended by the GATK v4.2.6.0 documentation. In the second part of the variant discovery process, the variant-called samples were consolidated, and genotyping was performed jointly for the whole cohort (Appendix C.2).

The workflow management software Snakemake 7.12.1 (accessed on 1 August 2022) was used to orchestrate the per sample preprocessing and variant calling part of the pipeline (Figure A2) [87].

Based on the preprocessing methods of Cucco et al. [73,74], the adapter-trimmed raw sequencing files were mapped to the Genome Reference Consortium Human Build 38 (GRCh38) human reference genome using the Burrow-Wheeler Aligner (bwa) v0.7.17 program in the ‘align_bwamem’ rule [88,89]. The resulting sequence alignment map (SAM) files were converted to binary alignment maps (BAM) (‘sam_to_bam’) and then sorted (‘sort_bam’) and indexed using the SAMtools version 1.16.1 program [77]. The mapping efficiency of the sorted BAM files was assessed using the command ‘samtools stats’.

Next, we used GATK v4.2.6.0 to carry out germline short-variant discovery in accordance with GATK best practices [71]. First, any duplicate reads that were derived from the same original DNA sample were marked using the MarkDuplicates tool (‘mark_duplicates’). This was followed by calculating (‘base_recalibrate’) and correcting any errors detected in the base quality scores (‘apply_bqsr’) using the BaseRecalibrator and ApplyBQSR tools, respectively. Following these preprocessing steps, the SNP and indel variants were called for each sample using the HaplotypeCaller tool in GVCF mode (‘variant_call’).

### Appendix C.2. Joint Genotyping Using GATK and Variant Annotation Using VCFtools and ANNOVAR

In the second part of the variant discovery process, the samples that had undergone variant calling were subjected to consolidation, followed by joint genotyping. Here, the samples were separated into CeD and control groups before consolidating the samples into a joint dataset.

The joint genotyping was carried out using GATK programs with default settings (Figure A3). First, the germline cohort data were created by consolidating the per sample genomic variant call format (GVCF) files created by the HaplotypeCaller tool as described above. The sample consolidation step was carried out by the GenomicsDBImport tool (‘consolidate_gvcfs’). The resulting cohort database was passed to the GenotypeGVCFs joint genotyping tool (‘jointcall_cohort’). Next, the raw variants were filtered in a two-step process. First, the variant quality scores on the log-odds scale (VQSLOD) were calculated using the VariantRecalibrator tool (‘variant_recalibration’). A filtering threshold was applied to these variant quality scores to produce a set of high-quality variant calls using the ApplyVQSR tool (‘applyvqsr’). The output of the above joint cohort processes was a recalibrated VCF file that contained all genotyping data of the cohort. The recalibrated VCF files were annotated by applying VCFtools v0.1.17 in frequency (‘run_vcffreq’), count (‘run_vcfcounts’), and comparison (‘compare_vcf’) mode [90]. Variant annotation was also carried out using the table_annovar program from ANNOVAR version 8 June 2020 with default settings (‘table_annovar’) [91].

## Appendix D. Detailed CNV Analysis and Burden Testing Protocols

### Appendix D.1. BTNL8*BTNL3 CNV Analysis

**Table A5:** The rs72494581 surrogate SNP was used to infer the CNV at the *BTNL8-BTNL3* region of chromosome 5.

rs72494581 Genotypes		Associated CNV at *BTNL8-BTNL3* Region of Chromosome 5
***TT***	Homozygous for reference allele	Full-length *BTNL8-BTNL3* region on both copies of chromosome 5
***CT***	Heterozygous	One copy has full-length *BTNL8-BTNL3* region One copy has BTNL8*BTNL3 deletion
***CC***	Homozygous for alternative allele	*BTNL8*BTNL3* deletion on both copies of chromosome 5

### Appendix D.2. Detailed Burden Testing Protocol

To summarise, qualifying variants within a gene were selected that had a low minor allele frequency or were predicted to be pathogenic. Any qualifying SNPs with more than two alleles, called multi-allelic sites, were split into SNPs with two alleles for the analysis: the reference allele and one of the alternative alleles. These variants are termed bi-allelic variants [53]. The disease risk burden, or the number of minor alleles, in the control and CeD cohorts was counted and compared. The burden testing was performed using dominant models and recessive models in TRAPD. The dominant model considers individuals as carriers for gene burden, if they have at least one qualifying variant from the selected sites within the gene, while the recessive model requires the presence of two or more variants to be labelled a carrier [53]. As gene burden is an additive value, the zygosity of the qualifying sites does not matter, only the number of qualifying variants. For example, in a gene with three qualifying sites, an individual who is homozygous for the alternate allele for one qualifying site carries the same amount of gene burden as an individual who is heterozygous for two of the qualifying sites. The analysis was modified from the one described by Guo [53], to adapt it to this cohort, as the original pipeline used an external control dataset.

The GATK processed sequences were subjected to further preprocessing before being burden tested with TRAPD, as recommended by Guo [53]. First, multi-allelic variants were separated using BCFtools version 1.16, as required by the TRAPD manual [77]. Next, the control and CeD cohort sequencing files were annotated using Ensembl Variant Effect Predictor (VEP) 109.3, and the SNPs were filtered to contain only non-synonymous coding variants [78]. The hybridisation capture sequencing files were then analysed after read depth filtering (Figure A4).

The cohort files were read depth filtered using VEP to select sites, where more than 90% of samples had a read depth coverage of >10. The final step of the preprocessing was to index and intersect the CeD and control sequencing files, to get the common SNPs between the two groups.

Following the preprocessing, the TRAPD code was applied using Python 2.7 to create the SNP file from the CeD and the control cohort sequencing files using ‘make_snp_file.py’, which contains the qualifying variants from each gene. Carriers of the qualifying SNPs from both the control and the CeD files were counted using the ‘count_cases.py’ file. The ‘burden.R’ code was modified to adapt it to the targeted sequencing cohort, as the original pipeline used an external database as the control, while this analysis uses the control sequences from the same cohort.

## Appendix E. Detailed Protocol Single-Variant Testing Analysis of Selected Butyrophilin SNPs in the UK Biobank

### Appendix E.1. HLA Genotyping in the UK Biobank Using the HLA Imputation Data

The HLA typing code using the UK Biobank HLA imputation data is available at https://gitlab.developers.cam.ac.uk/path/soilleux/soilleux-group/ced_butyrophilin_phd/-/tree/dropbox/ukbiobank_hla_typing/hla_imputation_only (accessed on 7 March 2025).

To summarise, the code used the HLA imputation values from data-field 22182. These values describe the likelihood of each *HLA* genotype, of which 14 were *HLA-DQA1* alleles and 18 were *HLA-DQB1* alleles. The *HLA* alleles were imputed by the UK Biobank from SNP data using the HLA*IMP:02 program [92]. In resource 182 (https://biobank.ctsu.ox.ac.uk/crystal/refer.cgi?id=182, accessed on 18 May 2022), the UK Biobank suggested using a threshold value of 0.7. If any *HLA* allele had an imputation value below 0.7, it was treated as an absent allele. The code applied this posterior threshold on the HLA imputation data for each participant, and the output was a list of *HLA* alleles that each participant had.

Afterwards, the CeD risk-associated *HLA* genotypes were called from the *HLA* allele data. The code calling CeD risk genotypes from the HLA imputation-derived alleles is available at https://gitlab.developers.cam.ac.uk/path/soilleux/soilleux-group/ced_butyrophilin_phd/-/blob/dropbox/ukbiobank_hla_typing/hla_imputation_only/ukbhla_fullcohort.ipynb (accessed on 2 September 2024).

To identify if a participant had CeD risk genotypes, the code looked for the presence of the risk alleles at the *HLA-DQA1* and *HLA-DQB1* loci. Participants who did not have alleles present at either locus were removed from the analysis. The participant was determined to have a CeD-associated *HLA* risk genotype if at least one copy of the risk *HLA-DQA1* and the *HLA-DQB1* alleles was present. If there were alleles present for more than one HLA risk genotype, the participant was typed as possessing both *HLA* risk genotypes.

### Appendix E.2. Detailed Single-Variant Testing of BTN2A1, BTN3A1, and BTN3A2 SNPs in the UK Biobank

As the SNPs were annotated using the dbSNP instead of their genomic position, and the dbSNP was updated to GRCh38 data at the time of this analysis, the SNP data could be used without further modification.

The individual SNP data in the UK Biobank were provided as the number of dbSNP reference alleles at each site, where 2 indicates that the individual is homozygous for the reference allele, while 1 indicates heterozygosity. If a participant had 0 reference alleles at a site, this could indicate homozygosity for the alternate allele or heterozygosity for two of the alternate alleles, depending on the number of potential alternate alleles. However, at the time of the analysis, the dataset did not provide information on which alternate allele was present.

To summarise the butyrophilin variant association analysis, firstly, the UK Biobank genome-wide genotyping dataset was curated and preprocessed for analysis. The downloaded per-chromosome UK Biobank genotyping data were loaded into R as a BEDMatrix object using the BGData 2.4.1 R package [80]. Afterwards, the genotype and phenotype data for the selected UK Biobank participants were merged. Next, data for all SNPs recorded in the *BTN2A1*, *BTN3A1*, *BTN3A2*, *BTNL3*, and *BTNL8* human genes were obtained from the National Centre for Biotechnology Information (NCBI) SNP database [65,81]. These SNPs were intersected with the genome-wide genotyping data, to identify the butyrophilin SNPs present in the UK Biobank dataset, which were 27 *BTN3A1*, 21 *BTN3A2*, 10 *BTNL3*, 13 *BTNL8*, and 30 *BTN2A1* SNPs. The final butyrophilin genotyping data in the UK Biobank dataset were provided as count data for the number of reference alleles at each SNP, identified by their rsIDs. Due to multiple testing, the resulting *p*-values were adjusted using Bonferroni correction.

Secondly, the association between butyrophilin variants and CeD risk was tested using binomial regression models, or binomial generalised linear models. In all of the linear models, the response variable was CeD status (CeD or no CeD, Table A6). The assumptions of the tests were that predictor variables were independent of each other. In the first test, the association between CeD risk *HLA* genotypes and CeD status was tested using one binomial model. In the second group of tests, individual binomial models were used to analyse the association between each butyrophilin family SNP and CeD risk. In the third group of tests, iterative binomial models were used that analysed the combined effect of butyrophilin family SNPs and *HLA* risk genotypes on CeD risk. In the fourth group of tests, the association between butyrophilin SNPs and CeD risk were analysed in *HLA*-matched groups.

**Table A6:** The binomial models tested the association between *HLA* risk genotypes and/or the individual butyrophilin family SNPs. Due to multiple testing, the resulting *p*-values were adjusted using Bonferroni correction. Abbreviations: CeD: coeliac disease; HLA: human leukocyte antigen; SNP: single-nucleotide polymorphism.

Test/Group Number	Association Being Tested	Predictor Variable(s)	Response Variable
**First test**	Association between *HLA* risk genotypes and CeD risk	*HLA* risk genotype	CeD status: CeD or control
**Second group of tests (101 models)**	Association between individual butyrophilin SNPs and CeD risk	Butyrophilin SNP: 2 reference alleles 1 reference allele 0 reference allele	CeD status: CeD or control
**Third group of tests (101 models)**	Association between the combined effect of *HLA* genotypes and butyrophilin SNPs and CeD risk	*HLA* risk genotype Butyrophilin SNP: 2 reference alleles 1 reference allele 0 reference allele	CeD status: CeD or control
**Fourth group of tests**	Association between butyrophilin SNPs and CeD risk in *HLA*-matched groups	Butyrophilin SNP: 2 reference alleles 1 reference allele 0 reference allele	CeD status: CeD or control

Thirdly, the risk ratio or odds ratio (OR), the *p*-value, and the 95% confidence intervals were calculated for each binomial model assessing the association between butyrophilin SNPs and CeD risk. The direction of each SNP was calculated from the natural logarithm of the OR values (ln(OR)). SNPs where ln(OR) < 1 indicated that the SNP decreased CeD risk. SNPs where ln(OR) > 1 indicated that the SNP increased CeD risk. Due to multiple testing, Bonferroni correction was applied for each group of tests.

The rsnps 0.5.0.0 R package was used to annotate the butyrophilin family SNPs in the UK Biobank significantly associated with CeD using the NCBI database [93]. The Hardy–Weinberg equilibrium of each SNP in the control group was assessed using the HardyWeinberg 1.7.7 R package [94].

## Appendix F. Detailed TRGV4 Usage and HV4 Amino Acid Sequence Analysis Pipeline

At the time of analysis, the software had a built-in reference library and could identify the clonotypes and gene segments used in the repertoire. The ‘analyze amplicon’ was a one-step command that aligned the sequencing data, assembled and exported the clonotypes found in the TCR repertoire. For the purposes of analysing the germline HV4 sequence, the region of interest was set to include the *FR3* of the *TRGV* genes (‘--region-of-interest {FR3Begin:CDR3End}’). The resulting text file contained the nucleotide sequence (‘targetSequences’), amino acid sequence (‘aaSeq’), clone count (‘cloneCount’), and V and J segment usage (‘allVHitsWithScore’, ‘allJHitsWithScore’) for each unique TRG sequence. The results from the MiXCR output were analysed using the pipeline available at https://gitlab.developers.cam.ac.uk/path/soilleux/soilleux-group/ced_butyrophilin_phd/-/tree/dropbox/trgv_hv4_analysis (accessed on 20 March 2025).

To identify the differences in the TRGV usage of the duodenal TRG repertoire, the read count of the TRGV section (‘allVHitsWithScore’) from the MiXCR output was processed using Python 3. Only samples from the duodenum were analysed for TRGV usage. Therefore, TRGV data from blood samples were not subjected to TRGV usage analysis.

## Appendix G. Supplementary Materials for Results Section 2.1

**Table A7:** CeD-associated *HLA* genotypes were found in 95.8% of CeD patients (n = 48) and 45.7% of controls (n = 46). (a) The CeD risk-associated *HLA* genotypes were called using HLA-HD [95]. (b) The *HLA-DQA1* and *HLA-DQB1* alleles of the two CeD patients, who did not have CeD risk-associated *HLA* genotypes. The *HLA-DQA1* allele of sample CD1 could not be typed by HLA-HD. CeD patients possessed a significantly higher proportion of risk *HLA* genotypes, when compared with controls in this dataset (Figure A6, Fisher’s exact test, *p* = 5.5 × 10^−10^).

(a)							
***HLA* Genotypes**	***HLA-DQ2.5***	***HLA-DQ8***	***HLA-DQ2.2***	***HLA-DQ2.5* and *DQ8***	***HLA-DQ2.5* and *DQ2.2***	***HLA-DQ8* and *DQ2.2***	**Other**
**CeD patients**	22	3	2	6	13	0	2
**Control participants**	10	2	6	0	1	2	25
(b)							
**Sample**	***HLA-DQA1* Allele**		***HLA-DQB1* Allele**		**Potential HLA-DQ Type**		
**CB26**	*HLA-DQA1*01:01:01*, *HLA-DQA1*02:01*		*HLA-DQB1*02:01:01*, *HLA-DQB1*05:01:01*		HLA-DQ5.1		
**CD1**	Not typed		*HLA-DQB1*02:01:01*, *HLA-DQB1*06:02:01*		Unknown		

**Table A8:** At least 47% of CeD patients (n = 48) and controls (n = 46) had the *BTNL8*BTNL3* deletion variant using the rs72494581 surrogate SNP. The *BTNL8*BTNL3* deletion variant encodes a truncated BTNL8-BTNL3 fusion protein [41]. Dart et al. [51] identified rs72494581 in the intronic region of *BTNL3*, which serves as a surrogate SNP, and the alleles are associated with the *BTNL8*BTNL3* copy number variant. The major allele, the *T* allele, is associated with the full-length *BTNL3* and *BTNL8* genes, while the minor allele, the *C* allele, is associated with the *BTNL8*BTNL3* deletion. The differences between the frequencies in CeD and control individuals failed to reach statistical significance (Figure A8, Fisher’s exact test, *p* = 0.2144).

rs72494581 Genotype	*TT*	*CT*	*CC*
***BTNL8-BTNL3* genes**	Homozygous for full-length sequence	Heterozygous for *BTNL8*BTNL3* deletion	Homozygous for *BTNL8*BTNL3* deletion
**Coeliac disease patients**	20	26	2
**Control participants**	24	17	5

**Table A9:** Less than 40% of sites found in CeD samples were shared with controls. Shared polymorphic sites between CeD and control samples in (a) the whole hybridisation capture dataset, (b) in non-synonymous coding sites, (c) in read depth-filtered non-synonymous coding sites. Read depth was defined as the number of sequence reads per site. Read depth filtering was applied as a quality control step. Sites where the read depth (dp) was more than 10, in more than 90% of the samples in each group, passed the read depth filter. Sites described above can be multi-allelic, meaning they may have more than one alternative allele.

(a)				
	**Number of Butyrophilin Family Sites**	**Sites Unique to Group**	**Shared Sites**	**Non-Matching Overlapping Sites**
**Coeliac**	1168	701	435	32
**Control**	769	302		
(b)				
	**Number of Butyrophilin Family non-Synonymous Coding Sites**		**Sites Unique to Group**	**Shared Sites**
**Coeliac**	108		79	21
**Control**	58		29	
(c)				
	**Number of Butyrophilin Family Non-Synonymous Coding Sites (>90% Samples in Group dp > 10)**		**Sites Unique to Group**	**Shared Sites**
**Coeliac**	60		54	6
**Control**	21		15	

Non-matching overlapping sites were defined as polymorphic sites, where the reference and/or alternate alleles identified in each group were different alleles. For example, a non-matching overlapping SNP would have the same base pair position on the chromosome with the same reference allele, but the alternate allele in one group is different from the other group’s. Comparisons were carried out using vcftools 0.1.17 [90].

**Table A10:** Percentage data of burden testing of butyrophilin variants in CeD samples against controls from the hybridisation capture dataset.

Gene	Qual. SNPs	CeD %(≥1 HET)	CeD %(≥2 HET)	CeD %(HOM ALT)	CeD Total Qual. allele Freq	Control %(≥1 HET)	Control %(≥2 HET)	Control %(HOM ALT)	Control Total Qual. Allele Freq	Dominant Model *p*-Value	Recessive Model *p*-Value
***BTN2A1***	**3**	**45.8**	**43.8**	**6.3**	**0.281**	**10.9**	**8.7**	**0.0**	**0.047**	**1.46 × 10^−5^**	**3.70 × 10^−8^**
*BTN3A2*	1	10.4	0.0	2.1	0.073	19.6	0.0	2.2	0.120	0.929	0.946
*ERMAP*	1	43.8	0.0	16.7	0.385	43.5	0.0	15.2	0.370	0.516	0.988

Burden testing was carried out on butyrophilin variants in CeD samples against controls from the dataset with read depth filtering. During read depth filtering, only sites where more than 90% of samples had a read depth coverage of >10 were selected. Percentage values in columns 3–5 and 7–9 show the percentage of individuals with each genotype within the CeD and the control groups of the dataset, respectively. Significant results are highlighted in bold. Abbreviations: CeD: coeliac disease; freq: frequency; HET: heterozygous; HOM ALT: homozygous for the alternative allele; N: number; qual.: qualifying; SNP: single-nucleotide polymorphism.

**Table A11:** Per sample genotype of participants at significant *BTN2A1* qualifying SNPs from the hybridisation capture cohort.

(a) CeD participants			
**Position**	**6:26463432**	**6:26468098**	**6:26468317**
**rsID**	**rs13195509**	**rs3734542**	**rs3734543**
CB22	hom alt	hom alt	hom alt
CB24	hom alt	hom alt	hom alt
CB26	het	het	het
CB27	het	het	het
CB29	het	het	het
CB31	het	het	het
CB32	hom ref	hom ref	hom ref
CB33	hom ref	hom ref	hom ref
CB34	het	het	het
CB35	het	het	het
CB36	het	het	het
CB37	het	het	het
CB38	het	het	het
CB39	hom ref	hom ref	hom ref
CB40	hom ref	hom ref	hom ref
CB41	hom ref	hom ref	hom ref
CB43	hom ref	hom ref	hom ref
CB44	hom ref	hom ref	hom ref
CB45	het	het	het
CB46	hom ref	hom ref	hom ref
CB48	hom ref	hom ref	hom ref
CB49	hom ref	hom ref	hom ref
CB50	hom ref	hom ref	hom ref
CB51	het	het	het
CB52	hom ref	hom ref	hom ref
CB53	het	het	het
CB54	het	het	het
CB55	het	het	het
CB56	hom ref	hom ref	hom ref
CB57	hom ref	hom ref	hom ref
CB58	het	het	het
CB59	hom ref	hom ref	hom ref
CB60	hom ref	hom ref	hom ref
CB61	het	het	het
CB62	het	het	het
CB63	hom alt	hom alt	hom alt
CB65	hom ref	hom ref	hom ref
CB67	het	het	het
CB69	het	het	het
CB70	hom ref	hom ref	hom ref
CD1	hom ref	het	hom ref
CD2	hom ref	hom ref	hom ref
CD3	hom ref	hom ref	hom ref
CD4	het	het	het
CD5	het	het	het
CD6	hom ref	hom ref	hom ref
CD7	hom ref	hom ref	hom ref
CD8	hom ref	hom ref	hom ref
(b) Control participants			
**Position**	**6:26463432**	**6:26468098**	**6:26468317**
**rsID**	**rs13195509**	**rs3734542**	**rs3734543**
NB11	hom ref	hom ref	hom ref
NB13	hom ref	hom ref	hom ref
NB14	hom ref	hom ref	hom ref
NB16	hom ref	hom ref	hom ref
NB19	hom ref	hom ref	hom ref
NB2	hom ref	hom ref	hom ref
NB20	hom ref	hom ref	hom ref
NB21	het	het	het
NB22	hom ref	hom ref	hom ref
NB25	hom ref	hom ref	hom ref
NB28	het	het	het
NB29	hom ref	hom ref	hom ref
NB3	hom ref	hom ref	hom ref
NB30	hom ref	hom ref	hom ref
NB31	hom ref	hom ref	hom ref
NB32	hom ref	hom ref	hom ref
NB34	hom ref	hom ref	hom ref
NB35	hom ref	hom ref	hom ref
NB37	hom ref	hom ref	hom ref
NB38	hom ref	hom ref	hom ref
NB39	hom ref	hom ref	hom ref
NB41	hom ref	hom ref	hom ref
NB42	hom ref	hom ref	hom ref
NB44	hom ref	hom ref	hom ref
NB46	hom ref	hom ref	hom ref
NB47	hom ref	hom ref	hom ref
NB48	hom ref	hom ref	hom ref
NB49	hom ref	hom ref	hom ref
NB5	hom ref	hom ref	hom ref
NB50	het	het	het
NB52	hom ref	hom ref	hom ref
NB56	hom ref	hom ref	hom ref
NB58	hom ref	hom ref	hom ref
NB68	hom ref	hom ref	hom ref
NB69	hom ref	hom ref	hom ref
NB7	hom ref	hom ref	hom ref
NB70	hom ref	hom ref	hom ref
NB71	hom ref	hom ref	hom ref
ND1	hom ref	hom ref	hom ref
ND10	hom ref	hom ref	hom ref
ND2	hom ref	hom ref	hom ref
ND5	het	het	het
ND6	hom ref	hom ref	hom ref
ND7	hom ref	hom ref	hom ref
ND8	hom ref	hom ref	hom ref
ND9	hom ref	het	hom ref

Abbreviations: het: heterozygous; hom alt: homozygous for the alternative allele; hom ref: homozygous for the reference allele.

## Appendix H. Demographic Data of the Selected UK Biobank Participants

Firstly, the ages at recruitment of the participants were analysed. The distribution of the age at recruitment of neither controls nor of CeD patients followed a normal distribution (Figure A8). The mean age at recruitment for controls was 62, which was significantly higher than that of CeD patients (t = 27.297, df = 3539.3, *p* < 2.2 × 10^−16^), whose average age at recruitment was 58.

Secondly, the sex of CeD and control participants in the UK Biobank was investigated (Figure A9). Interestingly, the proportion of female participants was significantly higher in the CeD group (64.8%, 2005/3094) than in the control group (40.4%, 12 010/29,762) (X-squared = 683.91, df = 1, *p* < 2.2 × 10^−16^).

Thirdly, the ethnic background of the UK Biobank participants was analysed. In both CeD and control groups, the majority of participants had White British backgrounds, at 91.8% (26 850/29,762) and 90.2% (2841/3094), respectively (Figure A10). In both groups, Irish and any other white background were the second and third most frequent ethnic backgrounds, respectively. When the ethnic background of CeD and control participants was compared (X-squared = 42.294, df = 21, *p* = 0.00384), no significant difference was present after Bonferroni correction for multiple testing (adjusted *p* > 0.05).

Finally, the dietary web questionnaire answers of CeD and control individuals were examined for any differences between the two groups. GFD was excluded from the statistical analysis of dietary differences between CeD and control individuals in the UK Biobank. Firstly, being on a GFD was one of the exclusion criteria for the control cohort, to exclude potential, undiagnosed CeD cases. Therefore, this diet would have zero occurrences in the control group. Secondly, CeD patients generally follow a GFD, as it is currently the only treatment for CeD [4]. This created a higher occurrence of GFD in CeD patients compared to controls selected from the UK Biobank dataset. The majority of UK Biobank participants did not adhere to any special diet, with 94.7% of controls (28,185/29,762) and 71.2% of CeD patients (2204/3094) reporting no special diet (Figure A11). A low-calorie diet was the most common special diet in controls (3.1%, 926/29,762).

A GFD was the most common special diet in CeD participants of the UK Biobank (22.2%, 686/3094), with an additional 3.6% (112/3094) of patients following a GFD in addition to other special diets. The second and third most common special diet in the CeD group was a combination of a GFD with a low-calorie diet, and a GFD with a lactose-free diet, at 1.5% (45/3094) and 1.3% (40/3094), respectively. When any variation in a GFD was excluded from the CeD group, the most common diet was a low-calorie diet (1.3%, 39/3094). Interestingly, 74.2% (2296/3094) of CeD participants did not follow a diet that excluded gluten.

When excluding a GFD from the analyses, the proportions of participants following the special diets within the control group were significantly different from the CeD group (X-squared = 23.303, df = 11, *p* = 0.01602). The lactose-free–low-calorie, vegan, lactose-free–low-calorie–vegetarian, lactose-free–vegetarian, and lactose-free–low-calorie–vegan diets were only found in the control group after excluding CeD patients adhering to a GFD. This could be due to CeD patients following the aforementioned diets in addition to being on a GFD (Table A12).

**Table A12:** The significant difference between the special diets of controls (n = 29,762) and CeD participants not on a gluten-free diet (n = 2296) may stem from CeD patients following multiple diets in addition to following a gluten-free diet.

			Without GFD		With GFD	
Diet	N Controls on Diet	% Controls on Diet	N CeD on Diet	% CeD on Diet (Out of 2296)	N CeD on Diet	% CeD on Diet (Out of 3094)
Lactose-free–low calorie	20	0.067	0	0	5	0.162
Vegan	15	0.050	0	0	1	0.032
Lactose-free–low calorie–vegetarian	2	0.007	0	0	1	0.032
Lactose-free–vegetarian	2	0.007	0	0	1	0.032
Lactose-free–low calorie–vegan	1	0.003	0	0	2	0.065

## Appendix I. Supplementary Materials for Results Section 2.2

**Table A13:** CeD-associated *HLA* genotypes were found in 92.4% of CeD (n = 3094) and 57.6% of control (n = 29,762) participants from the UK Biobank’s 500,000 genome-wide genotyping dataset. The *HLA* genotype of selected participants from the 500,000 genome-wide genotyping dataset was called using the HLA imputation values provided by the UK Biobank. CeD risk *HLA* genotypes were significantly more frequent in CeD patients compared to control participants (X-squared = 4062.5, df = 6, *p* < 2.2 × 10^−16^).

*HLA* Genotypes	*HLA-DQ2.5*	*HLA-DQ8*	*HLA-DQ2.2*	*HLA-DQ2.5* and *DQ8*	*HLA-DQ2.5* and *DQ2.2*	*HLA-DQ8* and *DQ2.2*	Other
CeD participants	1652	171	199	606	182	50	234
Control participants	6416	4203	4154	895	886	590	12,618

**Table A14:** Coefficients of the binomial regression model investigating CeD risk *HLA* genotypes as a predictor variable for CeD status in the UK Biobank dataset. Results of the binomial generalised linear model testing the association between CeD status and CeD risk-associated HLA genotypes in the UK Biobank dataset. Abbreviations: NA: not applicable; ns: not significant.

*HLA* Genotype	Coefficient Estimate	Standard Error	z Value	*p*-Value	CeD Risk
***HLA-DQ2.2, HLA-DQ2.5***	2.649	0.090	29.550	<2 × 10^−16^	Increase
***HLA-DQ2.2, HLA-DQ8***	0.570	0.164	3.474	5.13 × 10^−4^	Increase
***HLA-DQ2.5***	1.682	0.078	21.662	<2 × 10^−16^	Increase
***HLA-DQ2.5, HLA-DQ8***	1.456	0.109	13.352	<2 × 10^−16^	Increase
***HLA-DQ8***	−0.163	0.107	−1.533	0.125	ns
**Other *HLA* genotype**	−0.949	0.098	−9.677	<2 × 10^−16^	Decrease
**Constant**	−3.039	0.073	−41.872	<2 × 10^−16^	NA

**Table A15:** Single-variant analysis of butyrophilin SNPs and CeD status without taking the *HLA* loci into account using the UK Biobank dataset. SNPs significantly associated with CeD status are in bold. Bonferroni correction was applied due to multiple testing.

SNP Name	Gene	OR	Upper	Lower	Adjusted *p*-Value	ln(OR)
rs10484441_G	*BTN2A1*	1.138386	1.249955	1.038835	0.60789	0.129612
rs12660069_C	*BTN2A1*	1.106915	1.299592	0.948994	1	0.101577
**rs13195402_G**	***BTN2A1***	**0.397002**	**0.424644**	**0.371261**	**4.67 × 10^−158^**	**−0.92381**
**rs13195509_G**	***BTN2A1***	**0.424358**	**0.45231**	**0.398239**	**1.61 × 10^−151^**	**−0.85718**
rs13437351_G	*BTN2A1*	1.485538	1.91326	1.176198	0.14151	0.395777
**rs1407045_A**	***BTN2A1***	**1.314112**	**1.385827**	**1.246309**	**6.07 × 10^−22^**	**0.273161**
rs142951857_A	*BTN2A1*	1.391065	3.107641	0.723169	1	0.33007
rs143104579_G	*BTN2A1*	1.14068	1.405753	0.935552	1	0.131625
rs146399224_T	*BTN2A1*	10963.24	NA	0.003938	1	9.302303
rs148111655_G	*BTN2A1*	1.130613	2.537072	0.583563	1	0.12276
**rs2273558_A**	***BTN2A1***	**0.673125**	**0.711994**	**0.636428**	**1.69 × 10^−41^**	**−0.39582**
**rs2893856_T**	***BTN2A1***	**0.839708**	**0.911101**	**0.772766**	**0.0032259**	**−0.1747**
rs2893857_C	*BTN2A1*	1.142895	1.255236	1.042695	0.48083	0.133565
rs3734539_C	*BTN2A1*	4032.285	NA	0.007	1	8.302088
**rs3734542_G**	***BTN2A1***	**0.425283**	**0.453292**	**0.39911**	**8.59 × 10^−151^**	**−0.855**
**rs3734543_G**	***BTN2A1***	**0.430012**	**0.458993**	**0.402974**	**1.59 × 10^−140^**	**−0.84394**
**rs3799380_T**	***BTN2A1***	**0.577632**	**0.611726**	**0.545555**	**8.59 × 10^−77^**	**−0.54882**
**rs56296968_C**	***BTN2A1***	**0.54644**	**0.579504**	**0.515375**	**9.70 × 10^−89^**	**−0.60433**
**rs6456724_T**	***BTN2A1***	**0.838697**	**0.91004**	**0.771803**	**0.00287**	**−0.17591**
rs6907857_T	*BTN2A1*	1.433106	1.834039	1.140956	0.29414	0.359844
rs6911470_C	*BTN2A1*	1.472809	1.963726	1.132407	0.57829	0.387171
**rs6929846_T**	***BTN2A1***	**0.813898**	**0.875261**	**0.756001**	**3.60 × 10^−6^**	**−0.20592**
rs7773913_C	*BTN2A1*	1.433061	1.833981	1.14092	0.29439	0.359813
**rs7773938_C**	***BTN2A1***	**0.548927**	**0.582092**	**0.517765**	**1.15 × 10^−87^**	**−0.59979**
rs77870445_T	*BTN2A1*	1.219723	1.609943	0.942473	1	0.198624
rs9348718_A	*BTN2A1*	1.267016	1.454243	1.109411	0.06114	0.236664
rs9358943_C	*BTN2A1*	1.768069	31.86429	0.363137	1	0.569888
**rs9358944_A**	***BTN2A1***	**0.546443**	**0.579355**	**0.515512**	**1.55 × 10^−89^**	**−0.60432**
**rs9358945_A**	***BTN2A1***	**0.54552**	**0.578367**	**0.514649**	**4.37 × 10^−90^**	**−0.60602**
rs9461254_G	*BTN2A1*	1.464419	1.953756	1.125151	0.66799	0.381458
**rs10456045_G**	***BTN3A1***	**0.638826**	**0.674371**	**0.605202**	**2.97 × 10^−57^**	**−0.44812**
rs10807008_G	*BTN3A1*	1.091584	1.192289	1.001092	1	0.087629
rs12200782_C	*BTN3A1*	1.138999	1.250121	1.039809	0.56581	0.13015
rs12207930_C	*BTN3A1*	1.147602	1.241418	1.062201	0.05418	0.137674
rs12208447_C	*BTN3A1*	1.284779	1.624554	1.030908	1	0.250586
rs12214924_T	*BTN3A1*	1.147514	1.241076	1.062324	0.05283	0.137597
rs143476765_A	*BTN3A1*	1.108868	4.616795	0.396888	1	0.10334
rs144114619_T	*BTN3A1*	1.212854	2.147941	0.740604	1	0.192976
rs145059723_A	*BTN3A1*	1.412767	4.034311	0.629895	1	0.34555
rs1741738_A	*BTN3A1*	1.144367	1.242176	1.05573	0.11623	0.134851
rs17610161_G	*BTN3A1*	1.097034	1.199223	1.005306	1	0.09261
**rs1796520_C**	***BTN3A1***	**0.758646**	**0.800004**	**0.719297**	**2.40 × 10^−22^**	**−0.27622**
**rs3799378_A**	***BTN3A1***	**0.585559**	**0.61957**	**0.553499**	**2.92 × 10^−75^**	**−0.53519**
**rs3857549_C**	***BTN3A1***	**1.247408**	**1.401099**	**1.114594**	**0.01526**	**0.221068**
rs3902051_A	*BTN3A1*	1.090365	1.187959	1.002366	1	0.086513
**rs41266839_G**	***BTN3A1***	**0.396746**	**0.423498**	**0.37178**	**2.12 × 10^−168^**	**−0.92446**
**rs4609015_T**	***BTN3A1***	**1.151594**	**1.245578**	**1.066033**	**0.03817**	**0.141147**
rs4712990_C	*BTN3A1*	1.101764	1.203269	1.010539	1	0.096912
rs55676749_T	*BTN3A1*	1.13993	1.38368	0.948273	1	0.130967
rs56161420_G	*BTN3A1*	1.138909	1.230815	1.055142	0.09381	0.130071
**rs6900725_T**	***BTN3A1***	**1.149401**	**1.242804**	**1.064341**	**0.04333**	**0.139241**
**rs6912853_C**	***BTN3A1***	**1.16488**	**1.257236**	**1.080577**	**0.00785**	**0.152618**
**rs6920986_C**	***BTN3A1***	**1.148238**	**1.241884**	**1.062975**	**0.04995**	**0.138228**
rs6921148_T	*BTN3A1*	1.165176	1.411589	0.971033	1	0.152872
**rs742090_A**	***BTN3A1***	**0.759078**	**0.800536**	**0.719639**	**3.58 × 10^−22^**	**−0.27565**
rs7770214_G	*BTN3A1*	1.145779	1.239155	1.060758	0.06048	0.136085
rs80153343_G	*BTN3A1*	1.136899	1.439622	0.910733	1	0.128305
**rs11758089_T**	***BTN3A2***	**1.192878**	**1.288514**	**1.105674**	**0.00063**	**0.176369**
**rs12176317_A**	***BTN3A2***	**0.445307**	**0.474099**	**0.41837**	**6.72 × 10^−140^**	**−0.80899**
rs12194095_C	*BTN3A2*	1.118798	1.235914	1.015164	1	0.112255
**rs12199613_C**	***BTN3A2***	**0.67037**	**0.706851**	**0.635751**	**1.76 × 10^−47^**	**−0.39993**
rs12205731_G	*BTN3A2*	1.114755	1.232063	1.011029	1	0.108634
rs144016445_G	*BTN3A2*	81101.36	NA	217.0251	1	11.30346
**rs1977_A**	***BTN3A2***	**0.445697**	**0.474831**	**0.418455**	**1.17 × 10^−136^**	**−0.80811**
**rs1979_G**	***BTN3A2***	**0.44537**	**0.474171**	**0.418426**	**8.23 × 10^−140^**	**−0.80885**
**rs1985732_A**	***BTN3A2***	**0.63289**	**0.668226**	**0.599467**	**2.87 × 10^−59^**	**−0.45746**
**rs2073526_G**	***BTN3A2***	**0.74435**	**0.785579**	**0.705115**	**9.15 × 10^−25^**	**−0.29524**
rs35183513_G	*BTN3A2*	1.102861	1.203659	1.012184	1	0.097908
rs58367598_T	*BTN3A2*	1.234256	1.449383	1.057523	0.89091	0.210469
rs7765566_G	*BTN3A2*	1.269787	1.499163	1.083157	0.39847	0.23885
rs9104_G	*BTN3A2*	1.092904	1.187567	1.007255	1	0.088838
**rs9358934_G**	***BTN3A2***	**0.447824**	**0.476845**	**0.420678**	**2.34 × 10^−137^**	**−0.80335**
**rs9379855_T**	***BTN3A2***	**0.447682**	**0.47666**	**0.420575**	**8.85 × 10^−138^**	**−0.80367**
**rs9379858_T**	***BTN3A2***	**0.448449**	**0.477474**	**0.421299**	**3.19 × 10^−137^**	**−0.80196**
**rs9379859_C**	***BTN3A2***	**0.447843**	**0.476903**	**0.420662**	**5.37 × 10^−137^**	**−0.80331**
rs9379861_G	*BTN3A2*	1.225507	1.619363	0.945713	1	0.203355
**rs9393713_G**	***BTN3A2***	**0.442958**	**0.471602**	**0.416159**	**1.07 × 10^−141^**	**−0.81428**
**rs9393714_G**	***BTN3A2***	**0.443419**	**0.47214**	**0.416551**	**6.99 × 10^−141^**	**−0.81324**
rs186813312_C	*BTNL3*	0.103966	NA	NA	NA	−2.26369
rs199970076_G	*BTNL3*	0.544013	10.42476	0.087697	1	−0.60878
rs201534771_G	*BTNL3*	0.108957	NA	NA	NA	−2.2168
rs201813197_C	*BTNL3*	1.141842	4.74926	0.409599	1	0.132642
rs35157246_C	*BTNL3*	1.069831	1.242264	0.926052	1	0.067501
rs4700774_G	*BTNL3*	0.943446	0.999778	0.89061	1	−0.05822
rs59220426_C	*BTNL3*	1.006054	1.132599	0.896669	1	0.006036
rs73815153_G	*BTNL3*	1.009988	1.138564	0.899028	1	0.009938
rs7713324_A	*BTNL3*	1.004294	1.130736	0.895001	1	0.004284
rs7726604_C	*BTNL3*	1.004751	1.13121	0.895444	1	0.00474
rs112469887_G	*BTNL8*	1.084234	1.330225	0.893133	1	0.080874
rs113071395_G	*BTNL8*	0.867372	1.006007	0.751681	1	−0.14229
rs113534626_A	*BTNL8*	1.019956	1.206531	0.868252	1	0.019759
rs141492316_T	*BTNL8*	0.891806	1.095588	0.733483	1	−0.11451
rs145199317_A	*BTNL8*	0.907765	1.254932	0.673198	1	−0.09677
rs151174174_C	*BTNL8*	0.770441	0.932722	0.641625	0.63031	−0.26079
rs17704291_C	*BTNL8*	0.940216	0.996283	0.88763	1	−0.06165
rs200633883_C	*BTNL8*	0.311552	1.114968	0.108457	1	−1.16619
rs201214790_T	*BTNL8*	4044.713	NA	1.08 × 10^−7^	1	8.305166
rs201891387_G	*BTNL8*	0.62355	2.663098	0.210953	1	−0.47233
rs2276995_A	*BTNL8*	0.983681	1.038422	0.931987	1	−0.01645
rs2619739_C	*BTNL8*	1.101246	1.212402	1.002386	1	0.096442
rs7724813_G	*BTNL8*	1.078621	1.169543	0.996169	1	0.075683

**Table A16:** SNP and allele count data for the significant SNPs from the non-HLA model. These SNPs were significantly associated with CeD status in single-variant testing of the UK Biobank dataset. The SNPs in bold remained significantly associated with CeD status in the binomial regression models that took the *HLA* genotype of individuals into account.

SNP, Reference Allele	Gene	Number of SNPs in Control	Number of SNPs in CeD	Total Allele Count in Control	Total allele Count in CeD	Total Number of SNPs in the UK Biobank	Total Allele Count in UK Biobank
**rs13195402_G**	***BTN2A1***	**52,060**	**4631**	**58,392**	**6028**	**56,691**	**64,420**
**rs13195509_G**	***BTN2A1***	**52,271**	**4654**	**59,474**	**6176**	**56,925**	**65,650**
rs1407045_A	*BTN2A1*	30,596	3603	59,306	6172	34,199	65,478
rs2273558_A	*BTN2A1*	34,599	3248	51,134	5570	37,847	56,704
rs2893856_T	*BTN2A1*	7815	697	59,452	6182	8512	65,634
**rs3734542_G**	***BTN2A1***	**52,209**	**4656**	**59,432**	**6180**	**56,865**	**65,612**
**rs3734543_G**	***BTN2A1***	**52,002**	**4644**	**59,146**	**6114**	**56,646**	**65,260**
rs3799380_T	*BTN2A1*	46,887	4213	59,354	6164	51,100	65,518
**rs56296968_C**	***BTN2A1***	**47,941**	**4295**	**59,422**	**6170**	**52,236**	**65,592**
rs6456724_T	*BTN2A1*	7813	696	59,418	6178	8509	65,596
rs6929846_T	*BTN2A1*	10,355	903	59,460	6180	11,258	65,640
rs7773938_C	*BTN2A1*	47,953	4289	59,472	6162	52,242	65,634
**rs9358944_A**	***BTN2A1***	**47,929**	**4294**	**59,462**	**6182**	**52,223**	**65,644**
**rs9358945_A**	***BTN2A1***	**47,944**	**4292**	**59,478**	**6182**	**52,236**	**65,660**
rs10456045_G	*BTN3A1*	41,458	3680	59,434	6174	45,138	65,608
rs1796520_C	*BTN3A1*	28,075	2504	59,240	6176	30,579	65,416
**rs3799378_A**	***BTN3A1***	**45,113**	**4017**	**59,206**	**6152**	**49,130**	**65,358**
rs3857549_C	*BTN3A1*	55,572	5848	59,430	6170	61,420	65,600
**rs41266839_G**	***BTN3A1***	**52,985**	**4720**	**59,430**	**6178**	**57,705**	**65,608**
rs4609015_T	*BTN3A1*	50,759	5371	59,452	6170	56,130	65,622
rs6900725_T	*BTN3A1*	50,682	5378	59,392	6182	56,060	65,574
rs6912853_C	*BTN3A1*	50,145	5329	59,434	6176	55,474	65,610
rs6920986_C	*BTN3A1*	50,787	5381	59,464	6182	56,168	65,646
rs742090_A	*BTN3A1*	28,172	2506	59,438	6174	30,678	65,612
rs11758089_T	*BTN3A2*	50,176	5354	59,432	6182	55,530	65,614
**rs12176317_A**	***BTN3A2***	**51,604**	**4597**	**59,488**	**6182**	**56,201**	**65,670**
**rs12199613_C**	***BTN3A2***	**36,321**	**3178**	**59,396**	**6178**	**39,499**	**65,574**
**rs1977_A**	***BTN3A2***	**50,506**	**4497**	**58,436**	**6074**	**55,003**	**64,510**
**rs1979_G**	***BTN3A2***	**51,551**	**4590**	**59,448**	**6176**	**56,141**	**65,624**
**rs1985732_A**	***BTN3A2***	**41,492**	**3674**	**59,442**	**6174**	**45,166**	**65,616**
rs2073526_G	*BTN3A2*	26,272	2288	59,434	6176	28,560	65,610
**rs9358934_G**	***BTN3A2***	**51,477**	**4591**	**59,412**	**6174**	**56,068**	**65,586**
**rs9379855_T**	***BTN3A2***	**51,458**	**4579**	**59,392**	**6162**	**56,037**	**65,554**
**rs9379858_T**	***BTN3A2***	**51,474**	**4586**	**59,422**	**6170**	**56,060**	**65,592**
**rs9379859_C**	***BTN3A2***	**51,530**	**4595**	**59,452**	**6174**	**56,125**	**65,626**
**rs9393713_G**	***BTN3A2***	**51,601**	**4590**	**59,472**	**6178**	**56,191**	**65,650**
**rs9393714_G**	***BTN3A2***	**51,581**	**4594**	**59,456**	**6180**	**56,175**	**65,636**

**Table A17:** The genotypes and Hardy–Weinberg equilibrium of the significant SNPs in the control participants from the non-HLA model. The Hardy–Weinberg equilibrium of each SNP in the control group was assessed using the HardyWeinberg R package [94]. Bonferroni correction was applied due to multiple testing.

SNP, Reference Allele	Gene	Number of Controls Homozygous for the Reference Allele	Number of Controls Heterozygous for the Reference Allele	Number of Control Individuals Without the Reference Allele	Allele Freq in Controls	HWE Adjusted *p*-Value
rs13195402_G	*BTN2A1*	23,188	5684	324	0.892	1
rs13195509_G	*BTN2A1*	23,018	6235	484	0.879	0.340
rs1407045_A	*BTN2A1*	7951	14,694	7008	0.516	1
rs2273558_A	*BTN2A1*	11,804	10,991	2772	0.677	0.179
rs2893856_T	*BTN2A1*	514	6787	22,425	0.869	1
rs3734542_G	*BTN2A1*	22,978	6253	485	0.878	1
rs3734543_G	*BTN2A1*	22,866	6270	437	0.879	1
rs3799380_T	*BTN2A1*	18,595	9697	1385	0.790	1
rs56296968_C	*BTN2A1*	19,326	9289	1096	0.807	1
rs6456724_T	*BTN2A1*	515	6783	22,411	0.869	1
rs6929846_T	*BTN2A1*	971	8413	20,346	0.826	1
rs7773938_C	*BTN2A1*	19,333	9287	1116	0.806	1
rs9358944_A	*BTN2A1*	19,319	9291	1121	0.806	1
rs9358945_A	*BTN2A1*	19,326	9292	1121	0.806	1
rs10456045_G	*BTN3A1*	14,460	12,538	2719	0.698	1
rs1796520_C	*BTN3A1*	6734	14,607	8279	0.526	1
rs3799378_A	*BTN3A1*	17,152	10,809	1642	0.762	1
rs3857549_C	*BTN3A1*	26,084	3404	227	0.935	1
rs41266839_G	*BTN3A1*	23,662	5661	392	0.892	1
rs4609015_T	*BTN3A1*	21,657	7445	624	0.854	1
rs6900725_T	*BTN3A1*	21,625	7432	639	0.853	1
rs6912853_C	*BTN3A1*	21,168	7809	740	0.844	1
rs6920986_C	*BTN3A1*	21,672	7443	617	0.854	1
rs742090_A	*BTN3A1*	6727	14,718	8274	0.526	1
rs11758089_T	*BTN3A2*	21,182	7812	722	0.844	1
rs12176317_A	*BTN3A2*	22,420	6764	560	0.867	1
rs12199613_C	*BTN3A2*	11,047	14,227	4424	0.612	1
rs1977_A	*BTN3A2*	21,828	6850	540	0.864	1
rs1979_G	*BTN3A2*	22,388	6775	561	0.867	1
rs1985732_A	*BTN3A2*	14,433	12,626	2662	0.698	1
rs2073526_G	*BTN3A2*	5874	14,524	9319	0.558	1
rs9358934_G	*BTN3A2*	22,330	6817	559	0.866	1
rs9379855_T	*BTN3A2*	22,327	6804	565	0.866	1
rs9379858_T	*BTN3A2*	22,329	6816	566	0.866	1
rs9379859_C	*BTN3A2*	22,358	6814	554	0.867	1
rs9393713_G	*BTN3A2*	22,422	6757	557	0.868	1
rs9393714_G	*BTN3A2*	22,404	6773	551	0.868	1

**Table A18:** Single-variant analysis of butyrophilin SNPs and CeD status in binomial regression models that took the *HLA* loci into account using the UK Biobank dataset. SNPs significantly associated with CeD status are in bold. Bonferroni correction was applied due to multiple testing.

SNP Name	Gene	OR	Upper	Lower	Adjusted *p*-Value	ln(OR)
rs10484441_G	*BTN2A1*	0.979307	1.082186	0.887707	1	−0.02091
rs12660069_C	*BTN2A1*	0.987937	1.171444	0.837975	1	−0.01214
**rs13195402_G**	***BTN2A1***	**0.812801**	**0.876887**	**0.753657**	**8.15 × 10^−6^**	**−0.20727**
**rs13195509_G**	***BTN2A1***	**0.824983**	**0.886694**	**0.767819**	**1.62 × 10^−5^**	**−0.19239**
rs13437351_G	*BTN2A1*	1.359939	1.781839	1.056236	1	0.30744
rs1407045_A	*BTN2A1*	1.061681	1.125065	1.001972	1	0.059854
rs142951857_A	*BTN2A1*	1.189393	2.744421	0.589877	1	0.173443
rs143104579_G	*BTN2A1*	1.045598	1.306706	0.844534	1	0.044589
rs146399224_T	*BTN2A1*	2678.29	NA	0.001107	1	7.892934
rs148111655_G	*BTN2A1*	1.06888	2.495456	0.522042	1	0.066611
rs2273558_A	*BTN2A1*	0.924046	0.983637	0.868186	1	−0.07899
rs2893856_T	*BTN2A1*	0.978844	1.068109	0.895906	1	−0.02138
rs2893857_C	*BTN2A1*	0.983276	1.0868	0.891136	1	−0.01687
rs3734539_C	*BTN2A1*	12320.17	NA	2.26 × 10^−5^	1	9.418993
**rs3734542_G**	***BTN2A1***	**0.828358**	**0.890318**	**0.770964**	**2.94 × 10^−5^**	**−0.18831**
**rs3734543_G**	***BTN2A1***	**0.845824**	**0.910556**	**0.785966**	**0.000823**	**−0.16744**
rs3799380_T	*BTN2A1*	0.906299	0.966317	0.850232	0.260718	−0.09839
**rs56296968_C**	***BTN2A1***	**0.889118**	**0.949206**	**0.833053**	**0.042016**	**−0.11753**
rs6456724_T	*BTN2A1*	0.975504	1.064451	0.892856	1	−0.0248
rs6907857_T	*BTN2A1*	1.296362	1.68837	1.012123	1	0.259562
rs6911470_C	*BTN2A1*	1.225626	1.666065	0.92177	1	0.203452
rs6929846_T	*BTN2A1*	0.956691	1.034347	0.884033	1	−0.04427
rs7773913_C	*BTN2A1*	1.29982	1.692843	1.014844	1	0.262226
rs7773938_C	*BTN2A1*	0.892443	0.95269	0.83623	0.062934	−0.11379
rs77870445_T	*BTN2A1*	0.971098	1.300178	0.738108	1	−0.02933
rs9348718_A	*BTN2A1*	1.100624	1.274443	0.954703	1	0.095877
rs9358943_C	*BTN2A1*	0.455962	8.266602	0.091903	1	−0.78535
**rs9358944_A**	***BTN2A1***	**0.888825**	**0.948639**	**0.833001**	**0.038293**	**−0.11786**
**rs9358945_A**	***BTN2A1***	**0.886765**	**0.946407**	**0.831099**	**0.02906**	**−0.12018**
rs9461254_G	*BTN2A1*	1.206876	1.642976	0.90642	1	0.188035
rs10456045_G	*BTN3A1*	0.918688	0.975087	0.865666	0.527112	−0.08481
rs10807008_G	*BTN3A1*	0.94089	1.033981	0.857412	1	−0.06093
rs12200782_C	*BTN3A1*	0.987553	1.090084	0.896183	1	−0.01253
rs12207930_C	*BTN3A1*	0.994811	1.081946	0.91566	1	−0.0052
rs12208447_C	*BTN3A1*	0.971864	1.246094	0.767755	1	−0.02854
rs12214924_T	*BTN3A1*	0.997777	1.08479	0.918712	1	−0.00223
rs143476765_A	*BTN3A1*	0.541997	2.391824	0.175983	1	−0.61249
rs144114619_T	*BTN3A1*	0.937876	1.708275	0.552209	1	−0.06414
rs145059723_A	*BTN3A1*	0.851224	2.534279	0.356178	1	−0.16108
rs1741738_A	*BTN3A1*	1.055187	1.152999	0.966854	1	0.053718
rs17610161_G	*BTN3A1*	0.948685	1.04337	0.863865	1	−0.05268
rs1796520_C	*BTN3A1*	0.925332	0.980491	0.873166	0.877114	−0.0776
**rs3799378_A**	***BTN3A1***	**0.866517**	**0.922476**	**0.814111**	**0.000704**	**−0.14327**
rs3857549_C	*BTN3A1*	1.20727	1.366146	1.0703	0.249929	0.188361
rs3902051_A	*BTN3A1*	0.947893	1.038919	0.865991	1	−0.05351
**rs41266839_G**	***BTN3A1***	**0.806793**	**0.868508**	**0.749711**	**1.06 × 10^−6^**	**−0.21469**
rs4609015_T	*BTN3A1*	0.999771	1.087084	0.920447	1	−0.00023
rs4712990_C	*BTN3A1*	0.953022	1.047148	0.8686	1	−0.04812
rs55676749_T	*BTN3A1*	1.055136	1.295119	0.867029	1	0.05367
rs56161420_G	*BTN3A1*	1.003319	1.090026	0.92446	1	0.003313
rs6900725_T	*BTN3A1*	0.998519	1.085343	0.919613	1	−0.00148
rs6912853_C	*BTN3A1*	1.060948	1.150125	0.979684	1	0.059162
rs6920986_C	*BTN3A1*	0.997232	1.08425	0.918167	1	−0.00277
rs6921148_T	*BTN3A1*	1.075901	1.317605	0.885986	1	0.073159
rs742090_A	*BTN3A1*	0.927391	0.982792	0.875004	1	−0.07538
rs7770214_G	*BTN3A1*	0.992737	1.079363	0.914026	1	−0.00729
rs80153343_G	*BTN3A1*	1.144379	1.465945	0.904679	1	0.134862
rs11758089_T	*BTN3A2*	1.093163	1.188249	1.00675	1	0.089075
**rs12176317_A**	***BTN3A2***	**0.820862**	**0.880647**	**0.765377**	**3.50 × 10^−6^**	**−0.1974**
rs12194095_C	*BTN3A2*	0.971352	1.079478	0.875824	1	−0.02907
**rs12199613_C**	***BTN3A2***	**0.884297**	**0.93714**	**0.834446**	**0.003312**	**−0.12296**
rs12205731_G	*BTN3A2*	0.970415	1.079025	0.874528	1	−0.03003
rs144016445_G	*BTN3A2*	38750.66	NA	151.7424	1	10.5649
**rs1977_A**	***BTN3A2***	**0.816781**	**0.87678**	**0.761121**	**2.06 × 10^−6^**	**−0.20238**
**rs1979_G**	***BTN3A2***	**0.820729**	**0.880499**	**0.765255**	**3.40 × 10^−6^**	**−0.19756**
**rs1985732_A**	***BTN3A2***	**0.896055**	**0.951463**	**0.84398**	**0.033488**	**−0.10975**
rs2073526_G	*BTN3A2*	0.925312	0.980986	0.872648	0.940603	−0.07762
rs35183513_G	*BTN3A2*	0.951537	1.044843	0.867784	1	−0.04968
rs58367598_T	*BTN3A2*	1.089293	1.290835	0.924221	1	0.085529
rs7765566_G	*BTN3A2*	1.145085	1.363507	0.967785	1	0.135479
rs9104_G	*BTN3A2*	0.945089	1.032973	0.86575	1	−0.05648
**rs9358934_G**	***BTN3A2***	**0.824601**	**0.884767**	**0.76877**	**7.53 × 10^−6^**	**−0.19286**
**rs9379855_T**	***BTN3A2***	**0.82361**	**0.883636**	**0.767905**	**6.04 × 10^−6^**	**−0.19406**
**rs9379858_T**	***BTN3A2***	**0.825671**	**0.885867**	**0.769811**	**8.99 × 10^−6^**	**−0.19156**
**rs9379859_C**	***BTN3A2***	**0.824802**	**0.885058**	**0.768893**	**8.10 × 10^−6^**	**−0.19261**
rs9379861_G	*BTN3A2*	1.039521	1.399242	0.785634	1	0.03876
**rs9393713_G**	***BTN3A2***	**0.814157**	**0.873453**	**0.759123**	**9.27 × 10^−7^**	**−0.2056**
**rs9393714_G**	***BTN3A2***	**0.818022**	**0.877663**	**0.762671**	**2.08 × 10^−6^**	**−0.20087**
rs186813312_C	*BTNL3*	0.387143	NA	NA	NA	−0.94896
rs199970076_G	*BTNL3*	0.890096	19.02105	0.105649	1	−0.11643
rs201534771_G	*BTNL3*	0.373628	NA	NA	NA	−0.98449
rs201813197_C	*BTNL3*	0.906025	3.98319	0.295074	1	−0.09869
rs35157246_C	*BTNL3*	1.061269	1.243749	0.909647	1	0.059466
rs4700774_G	*BTNL3*	0.953121	1.014114	0.896074	1	−0.04801
rs59220426_C	*BTNL3*	0.964396	1.094724	0.852069	1	−0.03625
rs73815153_G	*BTNL3*	0.979858	1.113514	0.864825	1	−0.02035
rs7713324_A	*BTNL3*	0.962434	1.092563	0.850278	1	−0.03829
rs7726604_C	*BTNL3*	0.963325	1.093541	0.851096	1	−0.03736
rs112469887_G	*BTNL8*	1.04641	1.299262	0.850631	1	0.045365
rs113071395_G	*BTNL8*	0.908391	1.065436	0.777899	1	−0.09608
rs113534626_A	*BTNL8*	1.008072	1.204841	0.848563	1	0.00804
rs141492316_T	*BTNL8*	0.930335	1.160408	0.752616	1	−0.07221
rs145199317_A	*BTNL8*	0.884686	1.250181	0.639686	1	−0.12252
rs151174174_C	*BTNL8*	0.828069	1.016412	0.679378	1	−0.18866
rs17704291_C	*BTNL8*	0.952291	1.013025	0.895481	1	−0.04888
rs200633883_C	*BTNL8*	0.419543	1.675223	0.123618	1	−0.86859
rs201214790_T	*BTNL8*	740.6942	NA	1.97 × 10^−8^	1	6.607588
rs201891387_G	*BTNL8*	0.495896	2.269513	0.147322	1	−0.70139
rs2276995_A	*BTNL8*	0.984754	1.043162	0.929755	1	−0.01536
rs2619739_C	*BTNL8*	1.078254	1.194765	0.974928	1	0.075343
rs7724813_G	*BTNL8*	1.054983	1.150319	0.968751	1	0.053525

**Table A19:** The genotypes and Hardy–Weinberg equilibrium of the significant SNPs in the control participants from the binomial models that took the *HLA* loci into account. The Hardy–Weinberg equilibrium of each SNP in the control group was assessed using the HardyWeinberg R package [94]. Bonferroni correction was applied due to multiple testing.

SNP, Reference Allele	Gene	Number of Controls Homozygous for the Reference Allele	Number of Controls Heterozygous for the Reference Allele	Number of Control Individuals Without the Reference Allele	Allele Freq in Controls	HWE Adjusted *p*-Value
rs13195402_G	*BTN2A1*	23,188	5684	324	0.892	1
rs13195509_G	*BTN2A1*	23,018	6235	484	0.879	0.184
rs3734542_G	*BTN2A1*	22,978	6253	485	0.878	0.246
rs3734543_G	*BTN2A1*	22,866	6270	437	0.879	1
rs56296968_C	*BTN2A1*	19,326	9289	1096	0.807	1
rs9358944_A	*BTN2A1*	19,319	9291	1121	0.806	1
rs9358945_A	*BTN2A1*	19,326	9292	1121	0.806	1
rs3799378_A	*BTN3A1*	17,152	10,809	1642	0.762	1
rs41266839_G	*BTN3A1*	23,662	5661	392	0.892	1
rs12176317_A	*BTN3A2*	22,420	6764	560	0.867	1
rs12199613_C	*BTN3A2*	11,047	14,227	4424	0.612	1
rs1977_A	*BTN3A2*	21,828	6850	540	0.864	1
rs1979_G	*BTN3A2*	22,388	6775	561	0.867	1
rs1985732_A	*BTN3A2*	14,433	12,626	2662	0.698	1
rs9358934_G	*BTN3A2*	22,330	6817	559	0.866	1
rs9379855_T	*BTN3A2*	22,327	6804	565	0.866	1
rs9379858_T	*BTN3A2*	22,329	6816	566	0.866	1
rs9379859_C	*BTN3A2*	22,358	6814	554	0.867	1
rs9393713_G	*BTN3A2*	22,422	6757	557	0.868	1
rs9393714_G	*BTN3A2*	22,404	6773	551	0.868	1

**Table A20:** Single-variant analysis of butyrophilin SNPs and CeD status using binomial regression models on the HLA-DQ2.5-matched case-control cohort of the UK Biobank database. SNPs significantly associated with CeD status are in bold. Bonferroni correction was applied due to multiple testing.

SNP Name	Gene	OR	Upper	Lower	Adjusted *p*-Value	ln(OR)
rs10484441_G	*BTN2A1*	1.026879	1.182655	0.894513	1	0.026524
rs12660069_C	*BTN2A1*	0.954382	1.207579	0.761803	1	−0.04669
**rs13195402_G**	***BTN2A1***	**0.757206**	**0.831328**	**0.689864**	**5.10 × 10^−7^**	**−0.27812**
**rs13195509_G**	***BTN2A1***	**0.77459**	**0.846648**	**0.708837**	**1.75 × 10^−6^**	**−0.25542**
rs13437351_G	*BTN2A1*	1.400742	2.090478	0.973921	1	0.337002
rs1407045_A	*BTN2A1*	1.080193	1.170756	0.996977	1	0.07714
rs142951857_A	*BTN2A1*	1.287748	4.431929	0.486536	1	0.252895
rs143104579_G	*BTN2A1*	1.331616	1.885897	0.963027	1	0.286393
rs146399224_T	*BTN2A1*	0.25741	NA	NA	NA	−1.35708
rs148111655_G	*BTN2A1*	0.944297	4.178925	0.294481	1	−0.05731
rs2273558_A	*BTN2A1*	0.892885	0.971388	0.820718	0.848901	−0.1133
rs2893856_T	*BTN2A1*	0.927404	1.048809	0.818043	1	−0.07537
rs2893857_C	*BTN2A1*	1.040565	1.199279	0.905854	1	0.039764
rs3734539_C	*BTN2A1*	27166.76	NA	9.99 × 10^−14^	1	10.20975
**rs3734542_G**	***BTN2A1***	**0.77697**	**0.849181**	**0.711073**	**2.52 × 10^−6^**	**−0.25235**
**rs3734543_G**	***BTN2A1***	**0.792317**	**0.867952**	**0.723463**	**5.43 × 10^−5^**	**−0.23279**
rs3799380_T	*BTN2A1*	0.869548	0.945573	0.799787	0.107572	−0.13978
**rs56296968_C**	***BTN2A1***	**0.852755**	**0.928301**	**0.783513**	**0.02331**	**−0.15928**
rs6456724_T	*BTN2A1*	0.924581	1.045479	0.815654	1	−0.07841
rs6907857_T	*BTN2A1*	1.401132	2.091061	0.974192	1	0.33728
rs6911470_C	*BTN2A1*	1.565436	2.679723	0.978088	1	0.448164
rs6929846_T	*BTN2A1*	0.870873	0.973832	0.777255	1	−0.13826
rs7773913_C	*BTN2A1*	1.408409	2.10183	0.979326	1	0.34246
**rs7773938_C**	***BTN2A1***	**0.854233**	**0.929778**	**0.784985**	**0.026611**	**−0.15755**
rs77870445_T	*BTN2A1*	1.022451	1.530146	0.704151	1	0.022203
rs9348718_A	*BTN2A1*	1.308813	1.636793	1.057849	1	0.26912
rs9358943_C	*BTN2A1*	0.257602	NA	NA	NA	−1.35634
**rs9358944_A**	***BTN2A1***	**0.850121**	**0.924966**	**0.781482**	**0.016059**	**−0.16238**
**rs9358945_A**	***BTN2A1***	**0.847905**	**0.922571**	**0.77943**	**0.012607**	**−0.16499**
rs9461254_G	*BTN2A1*	1.565001	2.678973	0.977818	1	0.447886
rs10456045_G	*BTN3A1*	0.872024	0.944198	0.805371	0.074344	−0.13694
rs10807008_G	*BTN3A1*	0.988264	1.128972	0.867514	1	−0.01181
rs12200782_C	*BTN3A1*	0.969241	1.112166	0.84719	1	−0.03124
rs12207930_C	*BTN3A1*	1.059407	1.19361	0.94228	1	0.05771
rs12208447_C	*BTN3A1*	1.183033	1.71485	0.838562	1	0.168081
rs12214924_T	*BTN3A1*	1.059617	1.192843	0.943249	1	0.057908
rs143476765_A	*BTN3A1*	0.385935	2.931895	0.063895	1	−0.95209
rs144114619_T	*BTN3A1*	0.800306	1.80142	0.391948	1	−0.22276
rs145059723_A	*BTN3A1*	1.546934	29.22579	0.264012	1	0.436275
rs1741738_A	*BTN3A1*	1.126398	1.288465	0.987689	1	0.119025
rs17610161_G	*BTN3A1*	0.999336	1.142965	0.876283	1	−0.00066
rs1796520_C	*BTN3A1*	0.901974	0.977599	0.831877	1	−0.10317
**rs3799378_A**	***BTN3A1***	**0.829346**	**0.900397**	**0.763968**	**0.00081**	**−0.18712**
rs3857549_C	*BTN3A1*	1.307821	1.557482	1.105147	0.217446	0.268362
rs3902051_A	*BTN3A1*	0.979611	1.113404	0.864043	1	−0.0206
**rs41266839_G**	***BTN3A1***	**0.753767**	**0.824792**	**0.68903**	**7.25 × 10^−8^**	**−0.28267**
rs4609015_T	*BTN3A1*	1.055122	1.187787	0.939241	1	0.053657
rs4712990_C	*BTN3A1*	1.009758	1.153906	0.886126	1	0.00971
rs55676749_T	*BTN3A1*	1.356015	1.867802	1.007293	1	0.30455
rs56161420_G	*BTN3A1*	1.060354	1.193295	0.944184	1	0.058603
rs6900725_T	*BTN3A1*	1.061349	1.194313	0.945175	1	0.059541
rs6912853_C	*BTN3A1*	1.087423	1.216203	0.974151	1	0.08381
rs6920986_C	*BTN3A1*	1.062734	1.196651	0.9458	1	0.060845
rs6921148_T	*BTN3A1*	1.340282	1.834522	1.000858	1	0.29288
rs742090_A	*BTN3A1*	0.903554	0.979423	0.833241	1	−0.10142
rs7770214_G	*BTN3A1*	1.053031	1.185369	0.937421	1	0.051673
rs80153343_G	*BTN3A1*	0.979216	1.347528	0.724804	1	−0.021
rs11758089_T	*BTN3A2*	1.191327	1.352007	1.052499	0.618146	0.175068
**rs12176317_A**	***BTN3A2***	**0.766628**	**0.836949**	**0.702371**	**2.82 × 10^−7^**	**−0.26575**
rs12194095_C	*BTN3A2*	0.943672	1.092386	0.818034	1	−0.05798
**rs12199613_C**	***BTN3A2***	**0.842231**	**0.911373**	**0.77819**	**0.002057**	**−0.1717**
rs12205731_G	*BTN3A2*	0.933998	1.081909	0.809117	1	−0.06828
rs144016445_G	*BTN3A2*	73914.07	NA	1.75 × 10^−6^	1	11.21066
**rs1977_A**	***BTN3A2***	**0.764904**	**0.835801**	**0.700168**	**2.99 × 10^−7^**	**−0.268**
**rs1979_G**	***BTN3A2***	**0.76816**	**0.838582**	**0.70381**	**3.63 × 10^−7^**	**−0.26376**
**rs1985732_A**	***BTN3A2***	**0.860169**	**0.932012**	**0.793857**	**0.023502**	**−0.15063**
rs2073526_G	*BTN3A2*	0.90252	0.979048	0.831612	1	−0.10256
rs35183513_G	*BTN3A2*	1.000653	1.140268	0.880486	1	0.000652
rs58367598_T	*BTN3A2*	1.153984	1.470445	0.915197	1	0.143221
rs7765566_G	*BTN3A2*	1.171754	1.497136	0.928192	1	0.158502
rs9104_G	*BTN3A2*	1.010674	1.145196	0.894134	1	0.010617
**rs9358934_G**	***BTN3A2***	**0.772404**	**0.843549**	**0.707423**	**8.85 × 10^−7^**	**−0.25825**
**rs9379855_T**	***BTN3A2***	**0.771306**	**0.842174**	**0.706564**	**6.76 × 10^−7^**	**−0.25967**
**rs9379858_T**	***BTN3A2***	**0.774407**	**0.845625**	**0.709352**	**1.18 × 10^−6^**	**−0.25566**
**rs9379859_C**	***BTN3A2***	**0.770242**	**0.841254**	**0.705384**	**6.31 × 10^−7^**	**−0.26105**
rs9379861_G	*BTN3A2*	0.987622	1.586757	0.639198	1	−0.01246
**rs9393713_G**	***BTN3A2***	**0.760996**	**0.830868**	**0.697149**	**1.06 × 10^−7^**	**−0.27313**
**rs9393714_G**	***BTN3A2***	**0.765074**	**0.835356**	**0.700859**	**2.25 × 10^−7^**	**−0.26778**
rs186813312_C	*BTNL3*	0.257602	NA	NA	NA	−1.35634
rs199970076_G	*BTNL3*	0.272926	6.904492	0.010788	1	−1.29856
rs201534771_G	*BTNL3*	0.273671	NA	NA	NA	−1.29583
rs201813197_C	*BTNL3*	0.514697	3.715301	0.100372	1	−0.66418
rs35157246_C	*BTNL3*	1.03751	1.287524	0.842551	1	0.036824
rs4700774_G	*BTNL3*	0.978837	1.065525	0.899706	1	−0.02139
rs59220426_C	*BTNL3*	1.017762	1.216405	0.856252	1	0.017606
rs73815153_G	*BTNL3*	1.02474	1.225825	0.861442	1	0.024438
rs7713324_A	*BTNL3*	1.018119	1.216841	0.856546	1	0.017957
rs7726604_C	*BTNL3*	1.020763	1.219953	0.85881	1	0.02055
rs112469887_G	*BTNL8*	1.002918	1.333287	0.765187	1	0.002914
rs113071395_G	*BTNL8*	0.93155	1.162634	0.752338	1	−0.07091
rs113534626_A	*BTNL8*	0.9371	1.185234	0.747845	1	−0.06496
rs141492316_T	*BTNL8*	0.963798	1.314391	0.718601	1	−0.03687
rs145199317_A	*BTNL8*	1.12544	1.912393	0.697448	1	0.118174
rs151174174_C	*BTNL8*	0.729764	0.953457	0.564223	1	−0.31503
rs17704291_C	*BTNL8*	0.969451	1.055155	0.891212	1	−0.03103
rs200633883_C	*BTNL8*	0.171372	1.035119	0.022558	1	−1.76392
rs201214790_T	*BTNL8*	0.256248	NA	NA	NA	−1.36161
rs201891387_G	*BTNL8*	0.171476	1.035747	0.022572	1	−1.76331
rs2276995_A	*BTNL8*	1.002057	1.084332	0.926283	1	0.002055
rs2619739_C	*BTNL8*	0.966968	1.107812	0.846435	1	−0.03359
rs7724813_G	*BTNL8*	1.041603	1.171988	0.927725	1	0.040761

**Table A21:** SNP and allele count of the SNPs significantly associated with CeD in UK Biobank participants with the *HLA-DQ2.5* genotype. These SNPs were significantly associated with CeD status in the *HLA-DQ2.5*-matched single-variant testing of the UK Biobank dataset.

Participants with the *HLA-DQ2.5* Genotype							
SNP, Reference Allele	Gene	Number of SNPs in Controls with *HLA-DQ2.5*	Number of SNPs in CeD with *HLA-DQ2.5*	Total Allele count in Control with *HLA-DQ2.5*	Total Allele Count in CeD with *HLA-DQ2.5*	Total Number of SNPs in the UK Biobank with *HLA-DQ2.5*	Total Allele Count in UK Biobank with *HLA-DQ2.5*
rs13195402_G	*BTN2A1*	9398	2256	12,514	3206	11,654	15,720
rs13195509_G	*BTN2A1*	9408	2265	12,820	3296	11,673	16,116
rs3734542_G	*BTN2A1*	9386	2266	12,808	3300	11,652	16,108
rs3734543_G	*BTN2A1*	9366	2265	12,722	3258	11,631	15,980
rs56296968_C	*BTN2A1*	8609	2107	12,798	3290	10,716	16,088
**rs7773938_C**	***BTN2A1***	**8606**	**2106**	**12,804**	**3290**	**10,712**	**16,094**
rs9358944_A	*BTN2A1*	8603	2108	12,818	3304	10,711	16,122
rs9358945_A	*BTN2A1*	8607	2106	12,818	3302	10,713	16,120
rs3799378_A	*BTN3A1*	8129	1959	12,768	3286	10,088	16,054
rs41266839_G	*BTN3A1*	9577	2298	12,820	3298	11,875	16,118
rs12176317_A	*BTN3A2*	9347	2242	12,826	3302	11,589	16,128
rs12199613_C	*BTN3A2*	6396	1515	12,802	3300	7911	16,102
rs1977_A	*BTN3A2*	9135	2197	12,574	3248	11,332	15,822
rs1979_G	*BTN3A2*	9330	2242	12,808	3302	11,572	16,110
rs1985732_A	*BTN3A2*	7353	1777	12,816	3294	9130	16,110
rs9358934_G	*BTN3A2*	9333	2240	12,810	3292	11,573	16,102
rs9379855_T	*BTN3A2*	9324	2239	12,802	3294	11,563	16,096
rs9379858_T	*BTN3A2*	9321	2242	12,804	3296	11,563	16,100
rs9379859_C	*BTN3A2*	9340	2244	12,806	3296	11,584	16,102
rs9393713_G	*BTN3A2*	9345	2237	12,812	3298	11,582	16,110
rs9393714_G	*BTN3A2*	9346	2241	12,818	3300	11,587	16,118

**Table A22:** The genotypes and Hardy–Weinberg equilibrium of the significant SNPs in the control participants from the HLA-DQ2.5 matched case-control models. The frequency of all the examined SNPs significantly differed from the Hardy–Weinberg equilibrium. The Hardy–Weinberg equilibrium of each SNP in the control group was assessed using the HardyWeinberg R package [94]. Bonferroni correction was applied due to multiple testing.

SNP, Reference Allele	Gene	Number of Controls Homozygous for the Reference Allele	Number of Controls Heterozygous for the Reference Allele	Number of Control Individuals Without the Reference Allele	Allele Freq in Controls	HWE Adjusted *p*-Value
rs13195402_G	*BTN2A1*	3353	2692	212	0.751	**2.65 × 10^−31^**
rs13195509_G	*BTN2A1*	3303	2802	305	0.734	**3.26 × 10^−20^**
rs3734542_G	*BTN2A1*	3290	2806	308	0.733	**3.69 × 10^−20^**
rs3734543_G	*BTN2A1*	3278	2810	273	0.736	**1.35 × 10^−26^**
rs56296968_C	*BTN2A1*	2737	3135	527	0.673	**2.65 × 10^−31^**
rs7773938_C	*BTN2A1*	2737	3132	533	0.672	**2.65 × 10^−31^**
rs9358944_A	*BTN2A1*	2734	3135	540	0.671	**2.65 × 10^−31^**
rs9358945_A	*BTN2A1*	2737	3133	539	0.671	**2.65 × 10^−31^**
rs3799378_A	*BTN3A1*	2446	3237	701	0.637	**2.65 × 10^−31^**
rs41266839_G	*BTN3A1*	3429	2719	262	0.747	**2.65 × 10^−31^**
rs12176317_A	*BTN3A2*	3267	2813	333	0.729	**2.65 × 10^−31^**
rs12199613_C	*BTN3A2*	1505	3386	1510	0.500	**2.65 × 10^−31^**
rs1977_A	*BTN3A2*	3172	2791	324	0.726	**2.65 × 10^−31^**
rs1979_G	*BTN3A2*	3260	2810	334	0.728	**2.65 × 10^−31^**
rs1985732_A	*BTN3A2*	1974	3405	1029	0.574	**2.65 × 10^−31^**
rs9358934_G	*BTN3A2*	3259	2815	331	0.729	**2.65 × 10^−31^**
rs9379855_T	*BTN3A2*	3257	2810	334	0.728	**2.65 × 10^−31^**
rs9379858_T	*BTN3A2*	3253	2815	334	0.728	**2.65 × 10^−31^**
rs9379859_C	*BTN3A2*	3263	2814	326	0.729	**2.65 × 10^−31^**
rs9393713_G	*BTN3A2*	3269	2807	330	0.729	**2.65 × 10^−31^**
rs9393714_G	*BTN3A2*	3267	2812	330	0.729	**2.65 × 10^−31^**

## Appendix J. Supplementary Materials for Results Section 2.3

**Table A23:** There were no significant differences in the TRGV usage of FFPE CeD (n = 45) and healthy control (n = 108) samples after Bonferroni correction was applied. Raw *p*-values from Mann–Whitney U (MWU) tests were adjusted using Bonferroni correction to account for false positives due to multiple testing.

	FFPE CeD (n = 45) vs. FFPE Healthy Control (n = 108)	
	Raw *p*-Value (MWU)	Adjusted *p*-Value
**TRGV2**	0.775	1
**TRGV3**	0.411	1
**TRGV4**	0.812	1
**TRGV5**	0.906	1
**TRGV5P**	0.684	1
**TRGV7**	0.566	1
**TRGV8**	0.382	1
**TRGV9**	0.070	0.70
**TRGV10**	0.248	1
**TRGV11**	0.025	0.25

**Table A24:** There were no significant differences in the HV4 distribution between healthy controls (n = 238) and CeD patients (n = 141). The reference amino acid sequence KYDTYGSTRKNLRMILR is noted as WT in the table. Sequences with amino acid substitutions are provided in full. Pairwise Fisher’s exact test with Bonferroni correction was applied on the different HV4 phenotypes in CeD and healthy control patients.

	141 CeD vs. 238 Healthy Control Samples	
	Raw *p*-Values (Fisher)	Adjusted *p*-Values
**WT vs. WT, KYDTYGSTRQNLRMIL**	0.3925	1
**WT vs. KYDTYGSTRQNLRMILR**	0.3392	1
**WT vs. KYDTYGSTRQNLRMILR, KYDTYGSTR_ELENDTA**	0.3668	1
**WT vs. WT, KYNTYGSTRKNLRMILR**	0.3668	1
**WT vs. KYDTYGNTRKNLRMILR, WT**	1	1
**WT vs. WT, KYDTYGSTRKSLRMILR**	0.6258	1
**WT vs. KYDTYGSTRKSLRMILR**	1	1
**WT vs. WT, KYDTYGSIRKNLRMILR**	0.3668	1
**WT, KYDTYGSTRQNLRMILR vs. KYDTYGSTRQNLRMILR**	0.1697	1
**WT, KYDTYGSTRQNLRMILR vs. KYDTYGSTRQNLRMILR, KYDTYGSTR_ELENDTA**	0.4524	1
**WT, KYDTYGSTRQNLRMILR vs. WT, KYNTYGSTRKNLRMILR**	0.4524	1
**WT, KYDTYGSTRQNLRMILR vs. WT, KYDTYGNTRKNLRMILR**	1	1
**WT, KYDTYGSTRQNLRMILR vs. WT, KYDTYGSTRKSLRMILR**	1	1
**WT, KYDTYGSTRQNLRMILR vs. KYDTYGSTRKSLRMILR**	1	1
**WT, KYDTYGSTRQNLRMILR vs. WT, KYDTYGSIRKNLRMILR**	0.4524	1
**KYDTYGSTRQNLRMILR vs. KYDTYGSTRQNLRMILR, KYDTYGSTR_ELENDTA**	0.25	1
**KYDTYGSTRQNLRMILR vs. WT, KYNTYGSTRKNLRMILR**	0.25	1
**KYDTYGSTRQNLRMILR vs. WT, KYDTYGNTRKNLRMILR**	1	1
**KYDTYGSTRQNLRMILR vs. WT, KYDTYGSTRKSLRMILR**	0.5165	1
**KYDTYGSTRQNLRMILR vs. KYDTYGSTRKSLRMILR**	1	1
**KYDTYGSTRQNLRMILR vs. WT, KYDTYGSIRKNLRMILR**	0.25	1
**KYDTYGSTRQNLRMILR, KYDTYGSTR_ELENDTA vs. WT, KYNTYGSTRKNLRMILR**	1	1
**KYDTYGSTRQNLRMILR, KYDTYGSTR_ELENDTA vs. WT, KYDTYGNTRKNLRMILR**	1	1
**KYDTYGSTRQNLRMILR, KYDTYGSTR_ELENDTA vs. WT, KYDTYGSTRKSLRMILR**	1	1
**KYDTYGSTRQNLRMILR, KYDTYGSTR_ELENDTA vs. KYDTYGSTRKSLRMILR**	1	1
**KYDTYGSTRQNLRMILR, KYDTYGSTR_ELENDTA vs. WT, KYDTYGSIRKNLRMILR**	1	1
**WT, KYNTYGSTRKNLRMILR vs. WT, KYDTYGNTRKNLRMILR**	1	1
**WT, KYNTYGSTRKNLRMILR vs. WT, KYDTYGSTRKSLRMILR**	1	1
**WT, KYNTYGSTRKNLRMILR vs. KYDTYGSTRKSLRMILR**	1	1
**WT, KYNTYGSTRKNLRMILR vs. WT, KYDTYGSIRKNLRMILR**	1	1
**WT, KYDTYGNTRKNLRMILR vs. WT, KYDTYGSTRKSLRMILR**	1	1
**WT, KYDTYGNTRKNLRMILR vs. KYDTYGSTRKSLRMILR**	1	1
**WT, KYDTYGNTRKNLRMILR vs. WT, KYDTYGSIRKNLRMILR**	1	1
**WT, KYDTYGSTRKSLRMILR vs. KYDTYGSTRKSLRMILR**	1	1
**WT, KYDTYGSTRKSLRMILR vs. WT, KYDTYGSIRKNLRMILR**	1	1
**KYDTYGSTRKSLRMILR vs. WT, KYDTYGSIRKNLRMILR**	1	1
